# Diagnosing pleural effusions using mass spectrometry-based multiplexed targeted proteomics quantitating mid- to high-abundance markers of cancer, infection/inflammation and tuberculosis

**DOI:** 10.1038/s41598-022-06924-y

**Published:** 2022-02-23

**Authors:** Aleksandra Robak, Michał Kistowski, Grzegorz Wojtas, Anna Perzanowska, Tomasz Targowski, Agata Michalak, Grzegorz Krasowski, Michał Dadlez, Dominik Domański

**Affiliations:** 1 Mass Spectrometry Laboratory, Institute of Biochemistry and Biophysics - Polish Academy of Sciences, Warsaw, Poland; 2 Mazovian Center of Pulmonary Disease and Tuberculosis Treatment, Otwock, Poland; 3 Department of Geriatrics, National Institute of Geriatrics, Rheumatology and Rehabilitation, Warsaw, Poland

**Keywords:** Lung cancer, Diagnostic markers, Proteomics, Respiratory tract diseases, Biochemistry, Cancer, Biomarkers, Oncology, Diseases, Infectious diseases, Tuberculosis

## Abstract

Pleural effusion (PE) is excess fluid in the pleural cavity that stems from lung cancer, other diseases like extra-pulmonary tuberculosis (TB) and pneumonia, or from a variety of benign conditions. Diagnosing its cause is often a clinical challenge and we have applied targeted proteomic methods with the aim of aiding the determination of PE etiology. We developed a mass spectrometry (MS)-based multiple reaction monitoring (MRM)-protein-panel assay to precisely quantitate 53 established cancer-markers, TB-markers, and infection/inflammation-markers currently assessed individually in the clinic, as well as potential biomarkers suggested in the literature for PE classification. Since MS-based proteomic assays are on the cusp of entering clinical use, we assessed the merits of such an approach and this marker panel based on a single-center 209 patient cohort with established etiology. We observed groups of infection/inflammation markers (ADA2, WARS, CXCL10, S100A9, VIM, APCS, LGALS1, CRP, MMP9, and LDHA) that specifically discriminate TB-PEs and other-infectious-PEs, and a number of cancer markers (CDH1, MUC1/CA-15-3, THBS4, MSLN, HPX, SVEP1, SPINT1, CK-18, and CK-8) that discriminate cancerous-PEs. Some previously suggested potential biomarkers did not show any significant difference. Using a Decision Tree/Multiclass classification method, we show a very good discrimination ability for classifying PEs into one of four types: cancerous-PEs (AUC: 0.863), tuberculous-PEs (AUC of 0.859), other-infectious-PEs (AUC of 0.863), and benign-PEs (AUC: 0.842). This type of approach and the indicated markers have the potential to assist in clinical diagnosis in the future, and help with the difficult decision on therapy guidance.

## Introduction

Pleural effusion (PE) is excess fluid in the pleural cavity often caused by cancer, most often lung adenocarcinoma (ADC), squamous cell carcinoma (SCC), small cell lung cancer (SCLC) and metastasis of other malignancies, and a variety of non-malignant diseases such as pneumonia (PN), tuberculosis (TB), heart and renal failure, and more than 30 other rare causes^1,2^. Effusions can be effectively distinguished, using the established Light’s criteria based on a simple pattern of biochemical markers derived from blood serum and pleural fluid, into either transudative, usually caused by systemic disease (e.g. heart/renal/hepatic failure), or exudative, usually caused by a pathology specific to the lung or pleura (e.g. cancer, TB, PN)^1^. However, exudate differentiation such as malignant from non-malignant effusions or parapneumonic from tuberculous is currently a diagnostic challenge due to similar biochemical and cellular profiles. For example, cancer and chronic inflammatory diseases such as TB generally have lymphocytic effusions, while PN fluid, and some TB-PE, are neutrophil-rich^2^. Determination of the PE etiology is a crucial step in diagnosis and for management of PEs but is now a time consuming process which can lead to unnecessary invasive procedures like thoracoscopy. PE fluid is often removed by less-invasive needle aspiration (thoracentesis), to ease dyspnea and for diagnostic purposes, and is an ideal source of potential disease markers due to the proximity to the pathology. Currently, only a few pathologies can be diagnosed from PE using clinical tests. While these have a very high specificity and are considered diagnostic, they often have a low sensitivity^2^. For example, sediment cytology, for identifying malignant PEs, often has a sensitivity below 60%, and pleural TB tests, like the gold-standard microbiological methods, take two weeks, and even with the PCR-based (Xpert MTB/RIF) test, are also insufficient with sensitivities below 23–63%^3,4^. Pleural infection has a high mortality and the gold standard for diagnosis, the growth of an organism in the PE, is negative in 40% of aspirates using standard culture and requires significant time^2^. Likewise, malignant-PEs and inflammatory effusions can have similar biochemical results. Recently, a large prospective study, assessing classic clinical parameters and markers, revealed six patient clusters for PEs caused mainly by cancer, TB, heart-failure, simple and complicated PN, empyema, and many other causes^5^. However, it showed that the same etiology could often cluster separately, while disparate etiologies could cluster together, highlighting the challenge of PE classification, and implying that a multiplexed marker panel would aid diagnosis. In recent years, many clinically used protein markers and those revealed by basic research, have been proposed to aid in the determination of PE etiology and thus better guide treatment decisions^4,6,7^.

Mass spectrometry (MS)-based targeted proteomics is on the cusp of entering clinical use and is undeniably the future of clinical protein-biomarker diagnostics^8,9^. MS is already routinely used in the clinic for small molecule quantitation and recently the measurement of peptides/proteins using targeted MS-based technologies is substituting for or complementing the older antibody-based techniques^10–12^. Targeted MS approaches (multiple/parallel reaction monitoring (MRM/PRM)) are able to overcome the many issues (cross-reactivity, patient autoantibodies, low multiplexing, expensive development etc.) of immunological assays which have been the “gold-standard” for protein quantitation^9,11^. MRM/PRM panels combined with isotope-labeled peptide standards provide a higher detail and specific biological information, are less expensive and quicker to develop, are characterized by higher analytical specificity and precision, a wider dynamic range, and the possibility of measuring numerous proteins within a single rapid analysis^8,9^. A fully-automated and certified MRM-MS-based clinical analyzer (Thermo Scientific™ Cascadion™) has recently been introduced for the analysis of small molecules^13^, and similar quantitation of protein disease markers in human bio-fluids is the probable next step^14,15^. We have therefore previously developed an MS-based targeted assay (MRM-panel) that quantitates multiple epithelial cytokeratin (CK) markers, currently used in pathological assessment of carcinomas, to aid in the determination of PE etiology. We have shown retrospectively that our assay can effectively differentiate malignant-PEs from non-malignant-PEs collected from patients in the clinic after routine thoracentesis^16^. Our less-invasive analysis of the PE fluid, as a ‘liquid-biopsy’ in contrast to invasive tumor excision or a biopsy of pleural tissue, could also differentiate patients with different lung cancer subtypes and could potentially shorten the time to diagnosis and guide treatment of patients that are inoperable^16,17^. Here, we expanded our MRM-panel assay to 53 proteins, with the addition of other established cancer-, TB- and infection/inflammation-markers that are currently individually assessed in the clinical laboratory, as well as by adding potential biomarkers suggested in the research literature for PE classification. These were also selected on the basis of being mid- to high-abundance (> 1 ng/mL) proteins in PEs to enable the easy future transfer of an uncomplicated sample preparation method (e.g. without enrichment or depletion) to a standard clinical LC–MS analyzer. A finalized MRM assay, consisting of a multiplexed panel of 34 markers, was used to analyze 209 patient PE samples with known etiology to assess the methods potential in classifying PEs towards either a benign, cancerous, TB or other-infection diagnosis. Using a multiclass classifier, we show a high discrimination ability for classifying these PE types, especially for distinguishing between the important cancerous-PEs and tuberculous-PEs. Furthermore, we are also able to discriminate the cancerous and infectious fluids from the benign-PEs, which encompass a wide variety of ailments that are often excluded in studies and which have, thus far, often lowered the specificity of a proposed assay or a biomarker^18,19^.

## Materials and methods

### Clinical PE specimens

PE samples were obtained during a standard thoracentesis procedure and were classified at the Mazovian Center of Pulmonary Disease and Tuberculosis Treatment (MCPDTT). The study was approved by the Bioethical Committee at the Regional Medical Chamber in Warsaw (KB/928/14) and by the Bioethical Committee of the National Institute of Geriatrics, Rheumatology and Rehabilitation in Warsaw (KBT-5/1/2019), and all research was performed in accordance with relevant guidelines and regulations. A written informed consent was obtained from each patient. Samples were deidentified and coded for the proteomic analyses and were handled according to Biosafety Level 2 practices. Three hundred and sixty-seven PE samples, from individual patients, were collected prospectively over a 5.7-year period and processed as described previously^16^, with the supernatants stored at -80 °C until analysis. We endeavored to collect most of the available PEs from the MCPDTT to obtain a real representation of the actual frequency of the different PE types encountered in such a single-center clinical setting. Effusions were classified as either transudative or exudative following Light’s criteria. Diagnosis of a malignant or cancer-related effusion was based on the demonstration of malignant cells in cytological examination of aspirated fluid or histopathological examination of pleural biopsy specimen, obtained by videothoracoscopy, performed by two experienced pathologists. Tuberculous pleuritis was diagnosed by confirmation of *Mycobacterium tuberculosis* in pleural fluid, sputum or bronchoalveolar lavage, or if pleural biopsy specimens obtained during videothoracoscopy revealed tuberculomas. Widely accepted clinical criteria were applied to diagnose other diseases. One hundred and fifty-eight PEs were excluded from the analysis (65 purulent empyema PEs, 55 PEs of unknown etiology, 8 PEs with two or more etiologies, and 30 benign-PEs as undefined transudate PEs—non-cancerous and non-infectious). Two hundred and nine PEs with a diagnosed etiology were taken for analysis. Makeup of the sample set and patient data are presented in Supplementary Table S1. One hundred and nine PEs were from patients with a diagnosed form of cancer (cancerous-PEs), and included 82 lung cancers (65 non-small-cell lung carcinomas (NSCLC: with 33 ADC and 22 SCC subtypes), and 17 SCLC types), and 27 secondary cancers (ten breast cancers, four pleural mesotheliomas, four ovarian cancers, and one of: malignant melanoma, lymphoma, leukemia, neuroendocrine cancer, kidney cancer, uterus cancer, bladder cancer, prostate cancer, and an unknown primary site). Positive cytology results were obtained in 23.6% of these cases. Fifty-eight PEs were from patients with infectious diseases (all-infectious PEs) and included 25 parapneumonic PEs, 24 tuberculous PEs (TB-PEs), 6 pleuritic PEs and 3 empyema PEs (non-purulent, i.e. no visual manifestation of pus). Forty-two PEs were from patients with non-cancerous and non-infectious ailments (benign-PEs), and included 27 heart failure-PEs, four transudates, five renal failure-PEs, two arthritis-PEs, and one of each PE related with: pancreatitis, cirrhosis, pulmonary embolism, and post-trauma.

### MRM assay development, optimization, and analysis

Proteotypic peptide selection for MRM analysis of each target protein, and optimization of peptide- and fragment-specific MRM settings using stable-isotope-labeled internal standard (SIS) peptides was performed as described previously^16^. Peptide selection was done manually and with the aid of the PeptidePicker software, using previously described criteria^20^. In brief, chosen proteotypic peptides met the following conditions: sequence uniqueness in the human proteome; numerous observations in spectral libraries; length not exceeding 21 amino acids; and, if possible, not containing easily chemically modifiable residues (e.g. Cys, Met) or sequences prone to modifications (e.g. DP, DG). Peptides were excluded when they had low digestion efficiency, a high frequency of single-nucleotide polymorphisms (SNPs), known posttranslational modifications (PTMs), or biological features affecting their measurement accuracy. The selected peptides were synthesized as SIS peptides using isotopically labeled amino acids on the C-terminus: Arg ^13^C_6_; ^15^N_4_ or Lys ^13^C_6_; ^15^N_2_ (98% isotopic enrichment) by JPT Peptide Technologies GmbH (Berlin, Germany) as SpikeTides_L. MRM analysis was conducted on a Waters Xevo triple-quadrupole (TQ) MS (Waters, Milford, MA) coupled to a Waters nanoAcquity UPLC via a Zspray Nanoflow source with a 10 μm SilicaTip PicoTip emitter (New Objective, MA, USA), a Waters nanoAcquity UPLC BEH130 C18 analytical column (75 μm × 150 mm, 1.7 μm particle size) at 40 °C, and a Waters Symmetry 100 C18 trap-column (180 μm × 20 mm, 5 μm particle size). All solvents and modifiers were LC–MS grade. Mobile phase A was 0.1% formic acid (Sigma-Aldrich) in water (J.T.Baker, Netherlands). Peptides were loaded onto the trap-column at 15 μL/min. and separated at 350 nL/min. using a 60-min LC run, with a gradient of acetonitrile (J.T.Baker) with 0.1% formic acid (mobile phase B) changing from 1 to 10% from 0 to 10 min and from 10 to 45% from 10 to 40 min, followed by a 90% B wash and equilibration. MS parameters included a capillary voltage of 3.5 kV, a purge gas flow of 100 L/h, cone gas flow of 5 L/h, NanoFlow gas set at 0.5 Bar, and a source temperature of 150 °C. The initial analytical methods targeted 53 proteins by scanning for 205 endogenous peptides, and 205 complementary SIS peptides, using optimized LC-MRM parameters. For increased specificity, the endogenous and SIS peptides were each monitored with five fragment ions (2050 transitions in total). The final method was reduced to primarily detectable proteins and targeted 34 markers with 105 peptides and SIS each, in two 60-min LC-MRM analyses per sample (818 transitions). Marker abundance in the PE sample is reported as a relative protein amount per volume of PE fluid. This is derived from the measured relative amount of one or more peptides (geometric mean) per protein, normalized to the respective SIS peptide(s). The quantity of an endogenous peptide is reported as the peak area ratio, being the sum of the peak areas of all transitions for the endogenous peptide divided by the sum of the peak areas of transitions of its heavy standard. To enable normalization of intensity data between samples in terms of MS signal fluctuations and post-digestion sample processing differences, equivalent SIS peptide amounts were added to all samples. Manual inspection of signals for each sample was made to ensure correct peak detection, accurate integration, and interference-free transitions. MRM methods and data analyses were performed in Skyline-Daily Ver. 21 (MacCoss Lab Software).

### PE sample in-solution digestion

PE samples were handled in a HEPA-filter-laminar safety cabinet prior to tryptic digestion and prepared using filter tips and ultrapure reagents (Sigma-Aldrich). Sample preparation and MRM analyses were randomized. After defrosting, PEs were centrifuged at 15,000 × g for 5 min. at 4 °C. PE fluid was analyzed on a per volume basis, taking 2.5 μL of each sample for analysis. Samples were denatured by adding 97.5 μL of 8.4 M urea in 50 mM Trizma-base/hydrochloride buffer (pH 8.4, Tris-buffer). They were next reduced with 2 μL of 50 mM tris(2-carboxyethyl)phosphine hydrochloride (TCEP) in 50 mM Tris-buffer for 30 min. at 37 °C, and then alkylated with 2 μL of 777 mM iodoacetamide in 50 mM Tris-buffer for 30 min. at 22 °C. Samples were incubated in this high urea concentration (7.88 M) at 37 °C for 4 h. Next, 741 μL of 50 mM Tris-buffer was added to decrease the urea concentration to 0.969 M, and proteins were digested with 4 μg (5 μL) of sequencing-grade modified trypsin (Promega # V5111, Fitchburg, WI) at 37 °C for 16 h. Samples were then incubated at 60 °C for 30 min., cooled to 37 °C, and a second addition of 4 μg (5 μL) of sequencing-grade modified trypsin was made, followed by an incubation for 2 h at 37 °C. A mixture of SIS peptides was added to give an amount of individual SIS ranging from 62.5 to 625 fmol/μg of digest, and samples were acidified with 0.1% formic acid (FA) to a final volume of 1.0 mL. Peptides were desalted using Oasis HLB 1-mL (10 mg) cartridges (Waters), which were primed with 1 mL LC–MS grade methanol, and 1 mL LC–MS grade water. Samples were loaded and washed with 1 mL of water, followed by elution in 200 μL of 65% LC–MS grade acetonitrile, 0.1% FA. Eluted samples were dried using a SpeedVac (Labconco), and re-suspended in 95 μL of 0.1% FA, sonicated for 5 min. and centrifuged at 14,000 × g for 3 min. For nano-LC–MS-MRM analysis, 3.8 μL of sample was injected. Two blanks (0.1% FA) were run between every sample, and a QC sample was regularly analyzed to monitor the performance of the LC–MS system.

### Statistical analysis

The data were processed using SPSS Statistics (Ver. 27.0; IBM Corp., Armonk, NY). The nonparametric Mann–Whitney U (MWU) test was performed for the protein level comparisons. Receiver operating characteristics (ROC) curves for pairwise comparisons were performed in SPSS, and the area under the curve (AUC) was reported to indicate diagnostic potential. Classification was performed within the Scikit-learn version 0.24.2 framework^21^. The classifiers were built using the XGBoost version 1.2.0 library^22^. Classifier performance was estimated using tenfold stratified cross-validation with hyper-parameter tuning performed in a nested fivefold cross-validation. The tuned hyper-parameters were: 'learning_rate', 'max_depth', 'min_child_weight', 'subsample', 'colsample_bytree', and 'n_estimators', with a full grid search performed. The cross-validation procedure was repeated 10 times with different random seed values. Data preprocessing was performed inside the cross-validation loop and involved scaling features to zero mean and unit standard deviation in the training set and missing value imputation. The imputation method used was k-nearest neighbors, with the default setting of k = 5. Recursive feature elimination was used for estimation of the optimal number of features needed for classification. Hyper-parameter grid size was reduced in this experiment due to high computational cost. In order to obtain a list of most important features for each group a separate model performing a one-vs. rest binary classification was trained. For comparison, a single split validation was performed with a stratified (PE-group proportions are same) random split of the data into 70%/30% train/test sets.

## Results

### Selection of proteins and development of a MRM panel assay

Fifty-three proteins (Table 1 and Supplementary Table S2) were selected for which specific MRM methods were built. These consist of markers currently used in the clinic, and putative markers proposed in the research literature, which have shown potential in the diagnosis or classification of PEs, of cancer samples, or disease from plasma/serum testing. Twenty-nine proteins represent clinical laboratory tests. Thirty-two, thirteen, and eight proteins are verified or putative cancer, TB, or infection/inflammation markers, respectively. One to seven peptides highly observable in proteomic databases (proteotypic), unique in sequence only to the target protein in the human proteome and devoid of modifiable amino acids, known PTMs, SNPs or biological features affecting their measurement accuracy, were selected for each marker to develop quantitative MRM methods, totaling 205 peptide targets. For each of these peptides, a synthetic stable-heavy-isotope-labeled internal standard (SIS) peptide was obtained to increase the assay’s specificity, precision, and sensitivity. MRM methods were empirically determined on these SIS peptides, and consist of peptide-specific optimized MS scan methods, targeting meticulously selected reporter peptides and their fragment ions, allowing to quantify markers with very high specificity and precision^16^ (Supplementary Table S3). For additional specificity and precision, 98% and 89% of the protein markers were targeted with two or more, and three or more peptides, respectively. SIS peptides were added in equal amounts to all samples allowing to normalize for sample processing variability post digestion and the LC–MS analysis step. The use of such standards fits the Tier 2 level definition established for MS-based targeted assays, which are precise, relative quantitative assays suitable for biomarker verification, and which can guide future developments of Tier 1 level assays suitable for clinical bioanalysis and diagnostic laboratory tests requiring absolute quantification^23^. Relative measurements of peptide levels, after normalization to SIS peptides and averaging of multiple peptides per protein, are used as surrogates to protein levels and are reported here as relative marker amounts per equal volume of PE fluid.

**Table 1:** Protein markers detected and measured in PE fluid out of fifty-three proteins assessed in this study. Tested proteins but not detected are described in Supplementary Table S2. These consist of markers currently used in the clinic, and putative markers proposed in the research literature, which have shown potential in the diagnosis or classification of PEs, of cancer samples, or disease from plasma/serum testing, using clinically established biochemical or antibody-based assays, or using research techniques such as global and targeted MS or antibody-based single- and multi-plexed methods. Twenty-nine proteins (ADA1 and ADA2 (representing ADA), C3, C9, CEA/CEACAM5, CK-6, CK-7, CK-8, CK-14, CK-17, CK-18, CK-19, CRP, GRP, IFNG, LDHA, LDHB, and LDHC (representing LDH), MSLN, MUC-1/CA 15–3, MUC-16/CA-125, NSE, CDH1, MMP9, EPCAM, VIM, WFDC2/HE4, SPB3/SCCA-1 and SPB4/SCCA-2 (representing SCCA)) represent clinical laboratory tests. Thirty-two, thirteen, and eight proteins are verified or putative cancer, TB, or infection/inflammation markers, respectively.

Protein name (abbreviation used in text) (other names) [gene name, UniProt identifier]	Peptide measured by MRM (Peptide position in protein. Bold indicates peptides used in final assay)	Reported concentration range in PEs	Description
**Cadherin-1 (CDH1)** (Epithelial cadherin, E-cadherin) [CDH1, P12830] Length: 882 aa, Mass (Da): 97,456	**NTGVISVVTTGLDR** (322–335) **GQVPENEANVVITTLK** (382–397) GLDARPEVTR (775–784)	408–872 ng/mL	Potential marker of malignant PE^6^. Clinical marker for differentiation of breast cancer^78^
**CD166 antigen (ALCAM)** [ALCAM, Q13740] Length: 583 aa, Mass (Da): 65,102	**YEKPDGSPVFIAFR** (58–71) **VLHPLEGAVVIIFK** (176–189) EGDNITLK (262–269) **ESLTLIVEGKPQIK** (406–419)	478–913 ng/mL	Potential marker of malignant PE^6^
**Hemopexin (HPX)** [HPX, P02790] Length: 462, Mass (Da): 51,676	**NFPSPVDAAFR** (92–102) LYLVQGTQVYVFLTK (318–332) **GGYTLVSGYPK** (333–343) **SGAQATWTELPWPHEK** (387–402)	NA	Potential marker of malignant PE^39,40^
**Keratin, type II cytoskeletal 6 (CK-6)** [KRT6A, P02538], Length: 564 aa, Mass (Da): 60,045, [KRT6B, P04259], Length: 564 aa, Mass (Da): 60,067, [KRT6C, P48668], Length: 564 aa, Mass (Da): 60,025	SGFSSVSVSR (KRT6A: 31–40) ATGGGLSSVGGGSSTIK (KRT6B: 534–550) **ADTLTDEINFLR** (KRT6 A,B,C: 288–299)	0–12 ng/mg protein	Potential marker of malignant PE^16^. Carcinoma tumor marker used in pathology^79^
**Keratin, type II cytoskeletal 7 (CK-7)** [KRT7, P08729] Length: 469 aa, Mass (Da): 51,386	LPDIFEAQIAGLR (137–149) **VDALNDEINFLR** (215–226) **FETLQAQAGK** (287–296)	0–24 ng/mg protein	Potential marker of malignant PE^16^. Carcinoma tumor marker used in pathology^79^
**Keratin, type II cytoskeletal 8 (CK-8)** [KRT8, P05787] Length: 483 aa, Mass (Da): 53,704	**LEGLTDEINFLR** (214–225) **YEELQSLAGK** (286–295) **LSELEAALQR** (353–362)	0–136 ng/mg protein	Potential marker of malignant PE^16^. Carcinoma tumor marker used in pathology and serological marker for therapy monitoring^79,80^
**Keratin, type I cytoskeletal 18 (CK-18)** [KRT18, P05783] Length: 430 aa, Mass (Da): 48,058	DWSHYFK (125–131) **AQIFANTVDNAR** (138–149) **AQYDELAR** (254–261)	0–42 ng/mg protein	Potential marker of malignant PE^16^. Carcinoma tumor marker used in pathology and serological marker for therapy monitoring^79,80^
**Keratin, type I cytoskeletal 19 (CK-19)** [KRT19, P08727] Length: 400 aa, Mass (Da): 44,106	**FGPGVAFR** (25–32) **AALEDTLAETEAR** (318–330) **SLLEGQEDHYNNLSASK** (382–398)	0–41 ng/mg protein	Potential marker of malignant PE^16^. Carcinoma tumor marker used in pathology and serological marker for therapy monitoring^79,80^
**Kunitz-type protease inhibitor 1 (SPINT1)** [SPINT1, O43278] Length: 529 aa, Mass (Da): 58,398	EGFINYLTR (135–143) TQGFGGSGIPK (154–164) **YTSGFDELQR** (372–381)	19–63 ng/mL	Potential marker of malignant PE^6^
**Mucin-1 (MUC1)** (Cancer antigen 15–3, CA 15–3) [MUC1, P15941] Length: 1,255 aa, Mass (Da): 122,102	**QGGFLGLSNIK** (1083–1093) **EGTINVHDVETQFNQYK** (1109–1125) **NYGQLDIFPAR** (1190–1200)	352–4140 ng/mL	Potential marker of malignant PE^6,37^. Clinical marker to monitor breast cancer therapy^35,36^
**Pigment epithelium-derived factor (SERPINF1)** [SERPINF1, P36955] Length: 418 aa, Mass (Da): 46,312	**LAAAVSNFGYDLYR** (54–67) **LQSLFDSPDFSK** (334–345) **DTDTGALLFIGK** (400–411)	NA	Potential marker of malignant PE^39^
**Sushi, von Willebrand factor type A, EGF and pentraxin domain-containing protein 1 (SVEP1)** [SVEP1, Q4LDE5] Length: 3,571 aa, Mass (Da): 390,170	**LLSDFPVVPTATR** (109–121) **GAFQQAAQILLHAR** (171–184) LLQTLETITNK (942–952)	537–1038 ng/mL	Potential marker of malignant PE^6^
**Thrombospondin-4 (THBS4)** [THBS4, P35443] Length: 961 aa, Mass (Da): 105,869	SSATIFGLYSSTDNSK (70–85) **AFAGPSQKPETIELR** (159–173) KPQDFLEELK (178–187) **NSLWHTGDTSDQVR** (851–864)	22–55 ng/mL	Potential marker of malignant PE^6^
**Galectin-1 (LGALS1)** [LGALS1, P09382] Length: 135 aa, Mass (Da): 14,716	SFVLNLGK (30–37) **DGGAWGTEQR** (65–74) **LPDGYEFK** (101–108)	NA	Potential marker of malignant pleural mesothelioma PE^69,70^
**Mesothelin (MSLN)** [MSLN, Q13421] Length: 630 aa, Mass (Da): 68,986	GLLPVLGQPIIR (250–261) EIDESLIFYK (310–319) **VNAIPFTYEQLDVLK** (339–353) IQSFLGGAPTEDLK (504–517) **TDAVLPLTVAEVQK** (536–549) **LLGPHVEGLK** (550–559)	6–40 nM	Potential marker of malignant pleural mesothelioma PE^42^. Clinical serum marker for monitoring of mesothelioma patients^41^
**Adenosine deaminase 2 (ADA2)** [ADA2, Q9NZK5] Length: 511 aa, Mass (Da): 58,934	TLIFPPSMHFFQAK (77–90) LLPVYELSGEHHDEEWSVK (249–267) **FVETHPEFIGIK** (276–287) LPYFFHAGETDWQGTSIDR (351–369) IGHGFALSK (382–390) **DIPIEVCPISNQVLK** (402–416)	NA	Clinically measured total ADA activity in PE is a marker of TB pleuritis, with ADA2 isoenzyme showing improved discrimination^3,51^
**Complement component C9 (C9)** [C9, P02748] Length: 559 aa, Mass (Da): 63,173	**TEHYEEQIEAFK** (214–225) TSNFNAAISLK (232–242) **LSPIYNLVPVK** (473–483) **AIEDYINEFSVR** (497–508)	NA	Potential serological marker of TB^45^. Clinical marker of inflammation
**C-X-C motif chemokine 10 (CXCL10)** (10 kDa interferon gamma-induced protein, IP-10) [CXCL10, P02778] Length: 98 aa, Mass (Da): 10,881	**CTCISISNQPVNPR** (30–43) **LEIIPASQFCPR** (48–59) VEIIATMK (60–67) CLNPESK (74–80)	670–4469 pg/mL	Potential marker of TB-PE^48,49^
**Gelsolin (GSN)** [GSN, P06396] Length: 782 aa, Mass (Da): 85,698	EVQGFESATFLGYFK (148–161) **HVVPNEVVVQR** (178–188) **QTQVSVLPEGGETPLFK** (374–390) **AGALNSNDAFVLK** (585–597) **TGAQELLR** (616–623)	NA	Potential marker of TB-PE^77^. Potential serological marker of TB^45^
**Kallistatin (SERPINA4)** [SERPINA4, P29622] Length: 427 aa, Mass (Da): 48,542	**IAPANADFAFR** (51–61) **VGSALFLSHNLK** (139–150) LFHTNFYDTVGTIQLINDHVK (167–187) FSISGSYVLDQILPR (321–335) **LGFTDLFSK** (336–344) ATLDVDEAGTEAAAATSFAIK (366–386)	NA	Potential serological marker of TB^45^
**Matrix metalloproteinase-9 (MMP9)** (Gelatinase B) [MMP9, P14780] Length: 707 aa, Mass (Da): 78,458	**AVIDDAFAR** (135–143) **AFALWSAVTPLTFTR** (144–158) **LGLGADVAQVTGALR** (604–618)	3–35 ng/mL	Potential marker of TB-PE, and clinical serum marker of inflammation^18,72,73^
**Tryptophan–tRNA ligase, cytoplasmic (WARS)** (Tryptophanyl-tRNA synthetase, TrpRS) [WARS, P23381] Length: 471 aa, Mass (Da): 53,165	**DLTLDQAYSYAVENAK** (205–220) **GIFGFTDSDCIGK** (265–277) **ISFPAIQAAPSFSNSFPQIFR** (278–298) **ALIEVLQPLIAEHQAR** (433–448)	NA	Potential serological marker of TB^45^
**Complement C3 (C3)** [C3, P01024] Length: 1,663 aa, Mass (Da): 187,148	**SNLDEDIIAEENIVSR** (749–764) **VHQYFNVELIQPGAVK** (1463–1478) **TFISPIK** (1583–1589)	NA	Potential marker of parapneumonic PE^39^ Clinical marker of inflammation
**C-reactive protein (CRP)** [CRP, P02741] Length: 224 aa, Mass (Da): 25,039	**ESDTSYVSLK** (32–41) **GYSIFSYATK** (66–75) **QDNEILIFWSK** (77–87)	3–105 mg/L	Potential marker of infectious PEs^58^. Clinical serum marker of infection and inflammation^57^
**Protein S100-A9 (S100A9)** [S100A9, P06702] Length: 114 aa, Mass (Da): 13,242	**NIETIINTFHQYSVK** (11–25) **LGHPDTLNQGEFK** (26–38)	NA	Potential marker of parapneumonic PE^39^
**Serum amyloid P-component (APCS)** [APCS, P02743] Length: 114 aa, Mass (Da): 13,242	**AYSLFSYNTQGR** (65–76) **VGEYSLYIGR** (87–96) **QGYFVEAQPK** (140–149)	NA	Potential marker of infectious PE^39^
**Vimentin (VIM)** [VIM, P08670] Length: 466 aa, Mass (Da): 53,652	**ILLAELEQLK** (130–139) FADLSEAANR (295–304) **ISLPLPNFSSLNLR** (411–424)	NA	Potential marker of infectious PE^16^. Tumor marker used in pathology^78^
**L-lactate dehydrogenase A chain (LDHA)** [LDHA, P00338] Length: 332 aa, Mass (Da): 36,689	**DYNVTANSK** (82–90) **FIIPNVVK** (119–126) **LLIVSNPVDILTYVAWK** (133–149) **VTLTSEEEAR** (306–315) **SADTLWGIQK** (319–328)	NA	Clinically used for exudative and transudative PE classification based on Light’s Criteria^1,76^. Clinical serum marker of inflammation^75^
**L-lactate dehydrogenase B chain (LDHB)** [LDHB, P07195] Length: 334 aa, Mass (Da): 36,638	LIAPVAEEEATVPNNK (8–23) **SLADELALVDVLEDK** (44–58) **IVVVTAGVR** (92–100) **GLTSVINQK** (300–308) **SADTLWDIQK** (320–329) **VIGSGCNLDSAR** (Common to LDH A, B, and C: 158/159–169/170)	NA	Clinically used for exudative and transudative PE classification based on Light’s Criteria^1,76^. Clinical serum marker of inflammation^75^

The sample preparation and protein digestion method was optimized to achieve an overall optimal assay sensitivity across the different markers in the panel, and to ensure that proteins are digested as close to completion as is technically possible. This was done due to the known variability in total protein concentrations in PEs and the potential effect of this on the tryptic digestion step which provides the surrogate reporter peptides. Consequently, this PE-specific in-solution digestion procedure consisted of a 4-h long urea denaturation step, and a high-heat incubation followed by a second addition of trypsin providing trypsin well in excess, to maximize protein denaturation and digestion, especially of hard to digest proteins. This also increased the assay’s level of sensitivity. Additionally, two abundant plasma proteins, serum albumin and transferrin, were monitored in all PE samples to indicate any overt problems with digestion. To determine which of the 53 markers are easily detectable with a standard TQ LC–MS instrument, the entire MRM panel was initially tested on a subset of pooled and individual samples encompassing the major PE etiologies: lung ADC and SCC, NSCLC, SCLC, mesothelioma, breast cancer, TB, pneumonia, and heart-failure. Based on these results, a final 34-marker panel of detectable and important proteins was selected, which quantitated 105 peptides and their corresponding SIS peptides, with 91% of markers targeted with two or more peptides. Next, we analyzed the 209 patient PEs using this marker panel, and were able to detect 29 proteins (Table 1). Levels of multiple peptides used per single protein, across the whole sample set, showed a median Pearson correlation coefficient (R value) of 0.926 ± SD 0.124, with the distribution shown in Supplementary Fig. S1, indicating overall a high agreement between the multiple peptides used, with some increased variability related to lower abundance markers. Peptides with overall very low-level signals were omitted in the final data analysis and those used are indicated in Table 1 and Supplementary Table S4.

### Analysis of 209 patient PEs and differences in abundance of specific protein markers in five PE types and their individual discrimination ability

For statistical analysis, the 209 PE samples were organized into five groups: cancerous-PEs (n = 109), all-infectious-PEs (n = 58), and benign-PEs (n = 42), with a further sub-division of the all-infectious-PEs into tuberculous-PEs (TB-PEs, n = 24), and other-infectious-PEs (n = 34). Table 2 presents proteins which were found to be at significantly different (p-value ≤ 0.05, MWU test) amounts between the five PE types, and their potential diagnostic utility as indicated by the AUC value from ROC curve analysis. These are shown in order of significance for all possible 26 comparisons, up to a p-value of ≤ 0.05 to enable a comparison of most markers, but we highlight those that performed best (p-value ≤ 0.01 in MWU-test, and AUC: ≥ 0.700). We analyze and report the levels of protein markers on a ‘per volume of PE’ basis as is commonly performed in the clinical laboratory for PE and other human bio-fluids. This is done despite the known variability in the total protein concentration amongst PEs, which in our case ranged as follows: all-PEs 41.2 g/L ± 9.31 SD, cancer-PEs 41.4 g/L ± 7.64 SD, TB-PEs 47.2 g/L ± 6.64 SD, other-infectious-PEs 47.8 g/L ± 5.36 SD, and benign-PEs 28.3 g/L ± 9.26 SD.

**Table 2:**
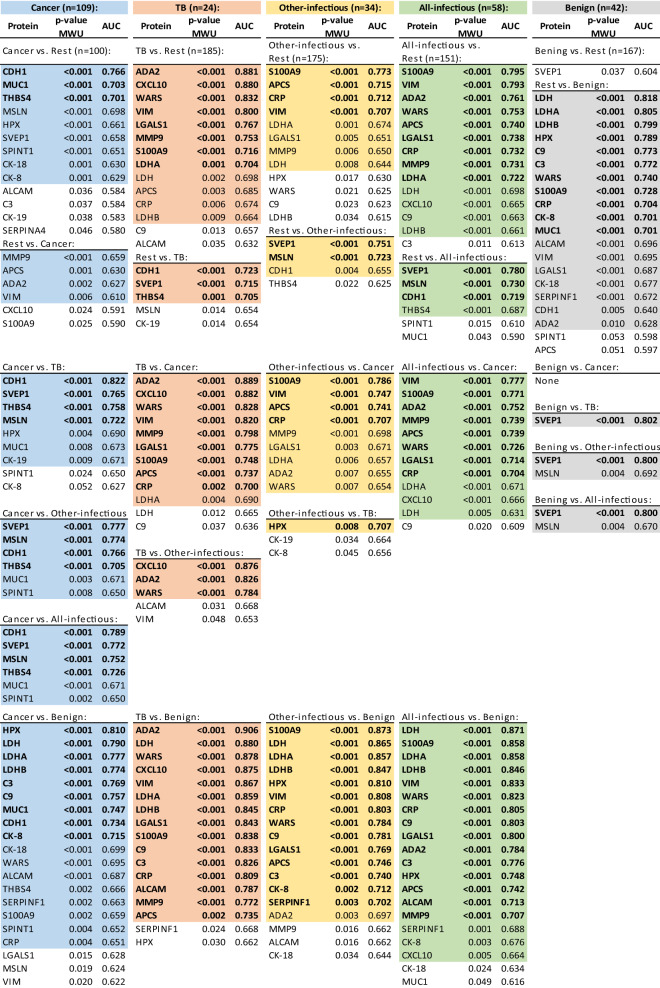
Protein markers significantly different between the five PE types, and their potential diagnostic utility. Shown in order of significance for all possible 26 comparisons as indicated by the AUC value from ROC curve analysis. All significant differences in marker amount with a p-value ≤ 0.05 (MWU test) are shown to enable a comparison of most markers, while those that performed best are highlighted (p-value ≤ 0.01 MWU test (colored), and AUC: ≥ 0.700 (bold)). For a detailed table including AUC 95% confidence intervals, significance values, and peptides used in the calculations see Supplementary Table S4.

**Figure 1:**
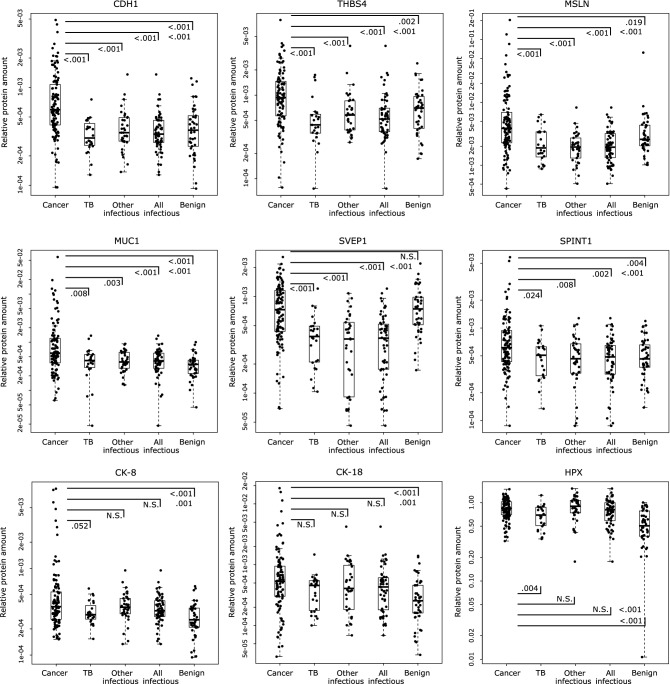
Proteins significantly elevated in the cancerous-PEs. Relative protein amounts in 209 patient PEs. Statistical significance (Mann–Whitney *U* test p-value) is indicated for the comparison of the cancerous-PEs vs. the TB-PEs, other-infectious-PEs, all-infectious PEs (TB-PEs and other-infectious-PEs combined), or benign-PEs (black lines), and vs. all other PEs combined (p-value between the all-infectious and benign PEs). Other MWU test results can be found in Table 2. Box-plots indicate the median and quartiles, with whiskers indicating the 1.5 interquartile range.

The cancerous-PE group had six prominent markers that were significantly elevated and comprised proteins involved in cell adhesion and cancer development: cadherin-1 (CDH1), thrombospondin-4 (THBS4), mesothelin (MSLN), mucin-1 (MUC1/CA 15-3), sushi von Willebrand factor type A EGF and pentraxin domain-containing protein 1 (SVEP1), and Kunitz-type protease inhibitor 1 (SPINT1) (Fig. 1). All six proteins could, individually, discriminate the cancerous-PE group from the all-infectious-PE group, as well as from the separated TB-PE and other-infectious-PE groups individually, and from all the non-cancerous PEs combined (n = 100), with an AUC ranging from 0.650 to 0.822. For this last comparison, CDH1, MUC1, THBS4, and MSLN were the most effective markers, with AUCs from 0.766 to 0.698. Overall, CDH1 was the best marker for effectively discriminating the cancerous-PEs in all five group comparisons (AUC: 0.734–0.822). In addition to CDH1, the proteins SVEP1, THBS4, and MSLN also effectively differentiated the cancerous-PEs from all three groups of infectious PEs (vs. TB, other-infectious, and all-infectious; AUCs: 0.705–0.777), but they were either not significant (SVEP1) or not as highly significant versus the benign-PEs. MUC1, as well as CK-8 and CK-18, which are clinically utilized cancer markers, were also significantly higher in the cancerous-PEs, although their discrimination ability was generally lower. However, comparing the cancerous-PEs to just the benign-PEs resulted in a higher AUC of 0.699–0.747 for these three markers. Of these, MUC1 performed the best, and could also modestly discriminate the cancerous-PEs from the three infectious-PE groups (AUC: 0.671–0.673). SPINT1 was significantly higher in the cancerous-PEs in all five group comparisons, however, its discrimination was lower. In summary, CDH1 is the best cancer-specific marker in this MS-based assay that can effectively discriminate the cancerous-PEs from each of the other PE groups. Additionally, THBS4, MSLN, and SVEP1 strengthen the differentiation of cancerous-PEs from the infectious-PEs, while MUC1 can also discriminate the cancerous-PEs from all non-cancerous PEs.

**Figure 2:**
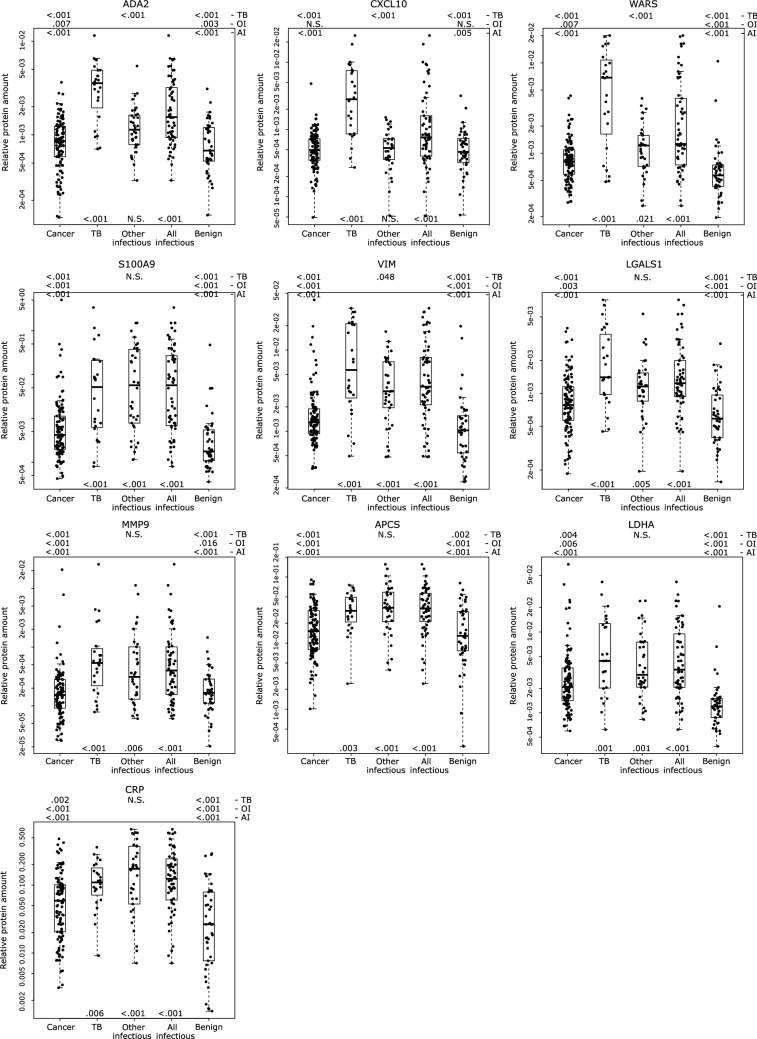
Proteins significantly elevated in the TB and infectious PEs. Relative protein amounts in 209 patient PEs. Statistical significance (Mann–Whitney *U* test p-value) is indicated above the graphs for the comparison of the TB-PEs (TB), other-infectious-PEs (OI), or all-infectious PEs (TB-PEs and other-infectious-PEs combined: AI) vs. cancerous-PEs, other-infectious-PEs, or benign-PEs (value above each group). Below the graphs are indicted p-values for the comparison of the TB-PEs, other-infectious-PEs, or all-infectious PEs vs. all other PEs combined. Other MWU test results can be found in Table 2. Box-plots indicate the median and quartiles, with whiskers indicating the 1.5 interquartile range.

The TB-PE group had three TB markers, adenosine deaminase 2 (ADA2), C-X-C motif chemokine 10 (CXCL10), and cytoplasmic tryptophan-tRNA ligase (WARS), that were highly elevated in only this group of PEs (Fig. 2). These were the only markers that could, with high significance, discriminate the TB-PEs from the other-infectious-PEs with an individual AUC ranging from 0.784 to 0.876. These were also the top markers differentiating the TB-PEs from all of the remaining non-TB PEs (n = 185) with AUCs of 0.832–0.881, and were within the top three and four markers discriminating TB-PEs from the cancerous- and benign-PEs (AUCs: 0.828–0.906), respectively. An additional seven proteins, elevated in the TB-PE group, also significantly discriminated it from the cancerous-, benign- and all non-TB-PEs, but these were also elevated in the other-infectious-PEs. These included mostly inflammation-related markers: vimentin (VIM), galectin-1 (LGALS1), serum amyloid P-component (APCS), protein S100-A9 (S100A9), matrix metalloproteinase-9 (MMP9), L-lactate dehydrogenase A chain (LDHA), and C-reactive protein (CRP), with AUCs from 0.674 to 0.867, and with VIM scoring highest. These seven proteins discriminated the other-infectious-PEs from the cancerous-PEs, benign-PEs and the rest of PEs (AUCs: 0.650–0.873), with S100A9 as the top marker in all three comparisons (AUCs: 0.773–0.873). Although the other-infectious-PEs did not have any elevated markers specific just to this group, hemopexin (HPX), which was also elevated in the cancerous-PEs (Fig. 1), was significantly higher in and discriminated this group from the TB-PEs (AUC: 0.707). Combining together the TB-PEs and the other-infectious-PEs into the all-infectious-PE group, also reveals the above ten proteins as being significantly elevated and each one being able to differentiate this infectious group from the cancerous-PEs, benign-PEs and from these PEs combined (AUCs: 0.664–0.858). In summary, the infectious PEs can be effectively distinguished by increased levels of ten markers. Seven of these, S100A9, VIM, LGALS1, APCS, MMP9, LDHA, and CRP, indicate an undefined ‘infectious PE’, but with S100A9 being a more effective other-infectious-PE marker. While the TB-PEs can be discerned with elevated levels of ADA2, CXCL10 and WARS, with ADA2 being the most effective marker with AUCs ranging from 0.826 to 0.906. ADA1, the other isoenzyme of total-ADA that is typically assessed in the clinic, was not detected in any of the analyzed samples.

Additional elevated markers were observed to be common between the above four disease groups (cancerous-, TB-, other-infectious- and all-infectious-PEs) and discriminated them from the benign-PEs (Supplementary Fig. S2). These included HPX, L-lactate dehydrogenase B chain (LDHB), LDH (encompasses LDH-A, -B and -C), complement C3 (C3), complement component C9 (C9), and pigment epithelium-derived factor (SERPINF1), with AUCs reaching up to 0.880. Half of these proteins are blood plasma proteins and this increase is likely due to the predominance of transudate PEs in the benign-PE group which are characterized by lower total plasma protein concentrations. This is further highlighted by many of the above mentioned disease-specific markers (e.g. WARS) being higher in each group than in the benign-PEs. However, SVEP1, which was indicated above as being increased in the cancerous-PEs compared to the infectious PE groups, was also the only marker found to be higher in the benign-PEs and was able to discriminate the benign-PEs from all three infectious PE groups (AUC: 0.800–0.802). Although no elevated markers were found to be specific only for the benign-PEs, a reduced level of the former six proteins and an increased SVEP1 concentration points towards a non-cancerous or non-infectious PE.

Other markers which were significantly elevated, although having a low discrimination ability, might also have future potential as disease markers in more sensitive MS-based assays. ALCAM, CK-19, and Kallistatin (SERPINA4), for example, were higher in cancerous-PEs (Supplementary Fig. S3), however, with low AUCs (0.580–0.584). Other proteins in our assay did not show a high significance in discriminating between the PE groups studied. CK-6 and CK-7 were detected in a few cancerous-PEs but their levels were not significantly different. Gelsolin (GSN) was detected with a good signal with four peptides in all of the samples, but no significant difference in protein levels was observed between any of the PE groups.

### Application of multi-class classifier for PE classification

We applied a machine learning methodology (Boosted Trees) to our data to reveal the most influential features and to show its capability in classifying patient PEs into one of the four etiology classes: cancerous, TB, other-infectious, or benign. An additional fifth group, all-infectious, was artificially created by merging the TB and other-infectious groups. We submitted abundance data in the form of the protein measurement as well as independent peptide levels to not miss a potentially better performing single peptide marker. The Boosted Trees method is based on sequential and progressive training of an ensemble of decision trees, where each tree added to the ensemble is constructed to improve the performance on previously misclassified instances. This confirms that the selected features have a powerful predicting capability. We used a tenfold stratified cross-validation repeated 10 times, effectively testing 100 random splits and averaging the outcomes. This allows to define the range of possibilities and provides standard deviation figures for estimated parameters. In addition, care was taken to avoid overfitting the model by choosing the hyperparameters in a nested cross-validation procedure.

**Figure 3:**
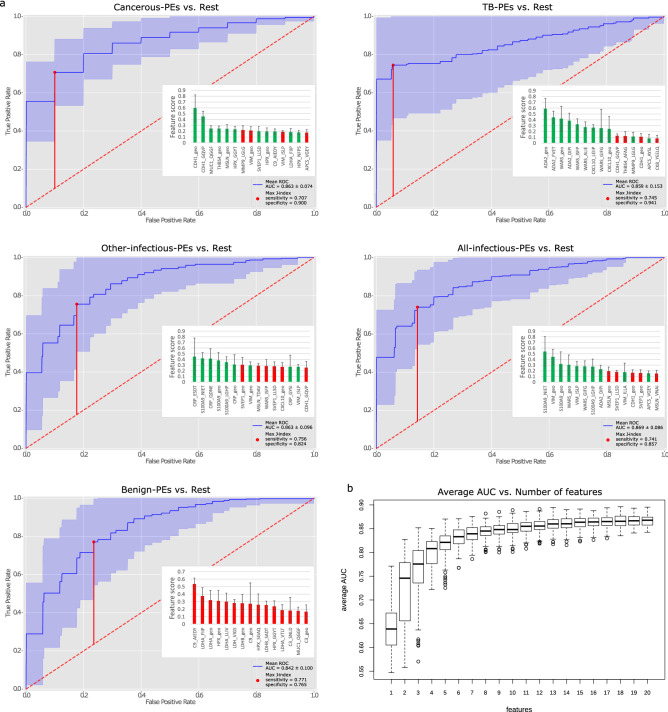
Performance of classifier at discriminating PEs into five etiology classes. (**a**) ROC curves showing diagnostic efficacy of classifier discriminating PEs into one of five etiology classes. Average AUC is displayed (blue line) with the ± standard deviation shaded (violet). An optimal %-sensitivity/%-specificity cut-off point is suggested by the maximum value of the Youden's index (vertical red line: Max J-index). For each classification, the 15 most influential features (individual protein peptide measurements or geometric means of multiple peptide values) used by the classifier are indicated (inset graph), with an indication if they had an increased (green) or decreased (red) level in the PE group. (**b**) Graph of average classifier AUC versus number of features used in total for the four classifications of cancerous-, TB-, other-infectious-, and benign-PEs.

The classifier was capable of predicting PE etiology with what is considered as a ‘very good’ discrimination ability^24^ for all five classes, with an AUC ranging from 0.842 to 0.869 (Fig. 3a). To suggest an optimal cut-off for a potential diagnostic tool based on this classifier we show the maximum value of the Youden's index (Max J-index), and report the sensitivity and specificity at that point. The top 15 features used for each of the classifications are displayed (Fig. 3a). The use of multiple markers by the classifier showed an improvement in discrimination ability as judged by the AUC, but especially by an increase in sensitivity at 100% specificity (zero false positive rate: 0 FPR). The prediction of cancerous-PEs and TB-PEs was especially effective, with a sensitivity of 55% and 67%, respectively, at a 0 FPR. The classifier discriminated the cancerous-PEs from the rest of PEs with an AUC of 0.863 ± 0.074, and with a 71% sensitivity and 90% specificity. The feature with the highest score was the level of CDH1, followed by lower-scoring elevated measurements for MUC1, THBS4, MSLN, and HPX. For the discrimination of TB-PEs an AUC of 0.859 ± 0.153 was obtained, with a 75% sensitivity and 94% specificity. The prominent top scoring features for this were a combination of increased peptide and protein levels for ADA2, WARS and CXCL10. The other-infectious-PEs were discriminated from the rest of PEs with an AUC of 0.863 ± 0.096, and with a 76% sensitivity and 82% specificity. The top six features were increased levels of CRP and S100A9, exclusively. We also tested the classifiers’ ability to predict the single larger all-infectious-PE class, resulting in an AUC of 0.869 ± 0.086, and a 74% sensitivity and 86% specificity. The top features were increased levels of S100A9, VIM, WARS, and ADA2. The classifier also had good discrimination ability for benign-PEs, which lacked any specific increased markers, with an AUC of 0.842 ± 0.100, and a 77% sensitivity and 77% specificity. The top two features for this were the decreased levels of C9 and LDHA, followed by 10 more features encompassing LDHA, C9, HPX, LDH, and LDHB. Cross-validation procedures such as the one used here are more rigorous for estimating classifier performance, especially when the dataset is relatively small, than classic single split validation techniques^25^. However, we also performed a training/testing approach by randomly splitting the 209 PEs into a 70%/30% stratified train/test ratio. The AUC results were very close to those from the cross-validation procedure, with three cases actually being higher: AUC-cancer = 0.833, AUC-TB = 0.924, AUC-other-infectious = 0.890, and AUC-benign = 0.851.

The minimum number of markers required in an assay, or the minimum number of single-marker assays needed to retain the same discriminatory ability, is an important consideration. Examining the effect of the number of total features used by the classifier for all four discriminations on the obtained AUC, reveals that using from 1 up to 15 features steadily increases the average AUC value from 0.645 to 0.862 (Fig. 3b). Increasing the number of features beyond 11 has a diminishing return on the AUC value, increasing from just 0.853 to 0.867 at 20 features. The use of 15 features appears to be a judicious choice, that would require the measurement of only 10 proteins (CDH1, MUC1, THBS4, ADA2, WARS, CRP, S100A9, C9, LDHA, and HPX), and is something that can be easily achieved in a single 30–60 min multiplexed LC–MS-MRM analysis.

### Clinical utility of assay-classifier

The trained model can be used for future classification of PEs based on the quantitative data obtained from this MRM panel analysis. The goal would be to use this as a supportive diagnostic tool, after standard imaging techniques and thoracentesis, to shorten PE diagnosis time and help in therapy guidance. We calculated the prevalence of the five PE etiologies used in the classifier based on the entire set of 367 collected PEs from this single-center patient cohort, while excluding purulent empyemas, which are easily diagnosed, and excluding PEs of unknown etiology (Table 3). This allows the calculation of clinically informative positive and negative predictive values (PPV and NPV, respectively), and we additionally show positive and negative likelihood ratios (LR + and LR-, respectively), and accuracy, at the chosen point of optimal sensitivity and specificity (maximal J-index) for each of the five classifications (Table 3). The classifier is best at discriminating cancerous-PEs and TB-PEs from the rest, with accuracies above 80%. The accuracy of classifying the other-infectious-PEs, all-infectious-PEs, and benign-PEs is slightly lower—between 76.8 and 79.9%. This is also seen in the likelihood ratios, where the LR + for TB-PEs is 12.6, indicating that a positive result significantly increases the probability that the patient has TB. For cancerous-PEs, the LR + is lower at 7.07, indicating a moderate-to-large improvement from the pretest probability (prevalence). The LR + for the other-infectious-PEs, all-infectious-PEs, and benign-PEs is lower (3.28–5.18), indicating a small-to-moderate improvement in our diagnostic ability for these PEs due to the test. The LR- for all PE groupings is similar, with a small-to-moderate decrease in probability after the test, indicating that a negative test result only modestly improves our ability to exclude PEs from a given group. The PPV/NPV values show that a classifier assignment of a cancerous-PE has a probability of 86% that the PE truly comes from a cancer patient, while a negative result indicates a 78% probability that it does not. In contrast, due to a lower prevalence, a classification to a TB-PE indicates a 58% likelihood of the PE originating from a TB patient, but a negative result indicates a 97% certainty it is not. A similar situation occurs for the other-infectious-PEs, all-infectious-PEs, and benign-PEs.

**Table 3:** Diagnostic performance of classifier with sensitivity/specificity cut-offs selected at the maximal J-index and taking the etiology prevalence into account.

Etiology	Prevalence %	% sensitivity	% specificity	PPV %	NPV %	LR +	LR-	Accuracy %
Cancerous-PEs	46.9	70.7	90.0	86.2	77.7	7.07	0.326	80.4
TB-PEs	9.8	74.5	94.1	57.8	97.1	12.6	0.271	84.3
Other-infectious-PEs	14.3	75.6	82.4	41.8	95.3	4.30	0.296	79.0
All-infectious-PEs	24.1	74.1	85.7	62.2	91.2	5.18	0.302	79.9
Benign-PEs	29.0	77.1	76.5	57.3	89.1	3.28	0.299	76.8

The choice of cut-off points (selected test sensitivity/specificity) can differ depending on the clinical question. In PE studies, these are often selected at higher specificity (low FPR) for a definite diagnosis, at the cost of missing positive cases. Inherently, a test like cytological analysis of PE, to diagnose malignancy, has 100% specificity. Setting the cut-off point in our classifier at 100% specificity, gives sensitivities of 55%, 67%, 40%, 48%, and 29% for identifying cancerous-, TB-, other-infectious, all-infectious-, and benign-PEs, respectively. Considering this approach further, we present curves of PPV vs. sensitivity for each of the five classifications, to allow one to determine the best possible cut-off point for a specific diagnostic question (Supplementary Fig. S4). Such curves can show where a test cut-off point could be made so as to not lose much in terms of sensitivity (i.e., to not miss positive cases) while obtaining a high PPV for a more definite diagnosis, while also taking the disease prevalence into account.

## Discussion

Targeted MS-based assays that use synthetic SIS peptides as internal standards have been shown to have unparalleled analytical specificity for the quantification of protein biomarkers, while being able to discern isoforms, PTMs, and add other biological insights^12,14,15^. This is in stark contrast to biochemical and antibody-based clinical assays whose specificity issues and limitations have recently been highlighted^10,11,26^. Although MS assays as ours, without marker-enrichment steps, lack the analytical sensitivity afforded by antibody-based assays, this is quickly changing due to instrumentation developments, and the adoption of MS-based protein assays in the clinical laboratory is undoubtedly the future^10,11,13–15^.

In this work, we determined which of the 53 currently used or putative protein markers previously suggested for determining PE etiology are the most likely to work in a targeted MS-based assay. These include approximately 20 mid- to high-abundance proteins which are easily detected with a simple sample preparation method, and make our approach easily transferable to a standard clinical LC–MS system. We show that a multiplexed approach with a multiclass classifier using up to 15 features improves the discrimination ability compared to using single markers. Recently, predicting PE etiology was shown to be a challenge due to the many potential causes, and likewise the assessment of multiple clinical biomarkers helped in clustering PEs^5^. Similar to our results, over half of malignant PEs clustered with transudate and infectious PEs, and infectious-PEs clustered often with TB and other PEs, while TB-PEs clustered mostly into a distinct group. Despite this challenge, we have shown that a MS-based approach has diagnostic merit. The AUC values for the five PE group classifications are between 0.842 and 0.869, with an average accuracy of correct diagnosis of 80%. The assay is very good at ruling-in a diagnosis of a TB-PE (LR + : 12.6), and second best, was its ability to rule-in a cancerous-PE diagnosis (LR + : 7.1). The ability of improving diagnoses of infectious and benign PEs was smaller. The rule-out ability for all of the PE groups was low, indicating that, in general, the absence of markers is not as good at indicating true negatives as the increase of marker concentrations is at indicating true positives. Such a test would therefore be best for differentiating an etiology of cancer or TB, which indeed is currently the biggest challenge, as both generally have lymphocytic effusions^2^. Sediment cytology, for identifying cancerous PEs, has a sensitivity below 60%, and pleural TB tests, like microbiological methods, take weeks, and even with the PCR-based tests, have sensitivities below 23–63%^3,4^. In our sample set, cytology results were positive for only 24% of the cancerous-PEs. In contrast, our assay (at 100% specificity), could identify 55% of the cancerous-PEs, and 67% of TB-PEs. We can conclude, based on this single-center PE cohort, that the diagnostic ability of our approach for these two etiologies is on par or better with the currently used clinical approaches, and may take less time.

A limitation of the presented results is that they are based on 209 PEs, with some PE types being of limited size. For this reason, a cross-validation procedure was used to predict the discrimination capabilities of a Boosted Trees classifier versus splitting this PE set into classic training/testing cohorts. A comparison of the cross-validation approach to randomly splitting the 209 PEs into 70%/30% train/test sets provided very similar results, indicating our approach is valid, and future analysis of larger independent cohorts could improve the ROC/AUC confidence ranges. At this exploratory stage of the markers, we also did not censor or modify values below the limit of detection (LOD) to avoid biased estimates of the AUCs^27,28^, and therefore, a future goal would be to define correct statistical methods to deal with such values, and/or to evolve this assay by using SIS peptides of defined quantity (e.g. by amino acid analysis) to clearly define quantitative limits as the LOD and lower limits of quantification (LLOQ), which would be especially necessary for clinical applications.

CDH1 was the best cancer-specific marker. THBS4, MSLN and SVEP1, in addition to CDH1, support the differentiation of cancerous-PEs from infectious PEs, including TB-PEs. MUC1, HPX, SPINT1, CK-8, and CK-18 could additionally discriminate the cancerous-PEs, with MUC1 being the most effective. The classifier used CDH1, MUC1, THBS4, MSLN, and HPX as the top features, in this order. HPX, however, was also elevated in the other-infectious PEs, and could discriminate these from the TB-PEs. SVEP1 was also the only marker significantly higher in and able to discriminate the benign-PEs from the infectious PEs. SPINT1, CK-8, and CK-18 were not among the top 15 features. This could be due to their lower significance or due to the classifier using a greedy algorithm to construct the trees, thus avoiding the use of redundant features. These markers might perform better with a more sensitive MS instrument or sample enrichment, as we have previously shown for the CK markers due to their lower abundance in PEs^16,17^.

CDH1 (E-cadherin), a component of adherens junctions, plays an important role in cell–cell adhesion of epithelial tissues. It is a serum biomarker candidate for the diagnosis of advanced colorectal cancer^29^. It was found to be elevated in malignant PEs, and was chosen as one of six top potential biomarkers in addition to SPINT1, MUC1, THBS4, ALCAM (CD166 antigen), and SVEP1^6^, which were all tested in our study. Our results generally corroborate the results of Chen et al., although with different discrimination abilities. Chen et al., used MRM on 82 PEs, but also used sample enrichment. They observed SPINT1 (AUC: 0.916), CDH1 (AUC: 0.815), and MUC1 (AUC: 0.807) as top markers allowing the discrimination of malignant PEs caused only by ADC. Furthermore, TB-PEs, PN-PEs, and para-malignant PEs (PEs associated with lung cancer, but without metastatic evidence) were used as controls. A logistic regression model obtained a best 3-marker panel, with SPINT1, SVEP1, and THBS4 (AUC: 0.951). Our study, in addition, included the benign-PEs (mostly transudates) which also show a higher level of SVEP1, making this marker not specific for cancerous-PEs, but rather indicating that it is lower in infectious-PEs. SVEP1 is an important cell adhesion molecule, regulating intercellular adhesion, with implications for carcinoma progression^30,31^. THBS4 is an extracellular calcium-binding protein involved in cell-to-cell and cell-to-matrix interactions, and was found to be overexpressed in cancer-associated stroma of diffuse-type gastric ADC secreted by cancer-associated fibroblasts, and in the stroma of invasive breast cancer^32,33^. MUC1, also known as cancer antigen 15–3 (CA 15–3), is a large transmembrane glycoprotein, expressed on the apical surfaces of most simple secretory epithelial cells, that undergoes auto-cleavage into two subunits (MUC1-N and MUC1-C)^34^, and is overexpressed in a variety of carcinomas including that of the lung^35^. Circulating MUC1-N, which includes the peptide assessed in our final assay, is used as a serological clinical marker (CA 15–3) to monitor breast cancer^36^. CA 15–3 measured clinically, within a panel of three other tumor marker tests (CEA, CA 125, and CYFRA 21–2 (CK-19); all targeted by our assay), was assessed for the diagnosis of malignant PEs, and when used alone, it had the best sensitivity, but only 30% (at 100% specificity). The combination of all four markers was reported to have reached a sensitivity of 54%^37^, which is similar to our classifier result of 55% (at 0 FPR). HPX is an abundant heme-binding plasma glycoprotein, with this important physiological role required to prevent oxidative damage during hemolysis^38^. It was higher in malignant PEs (ADC/NSCLC) compared to TB/PN-PEs^39,40^, but is also an acute phase reactant protein induced after inflammation^38^, and this might explain our observation of it also being elevated in the other-infectious-PEs. MSLN is normally expressed mostly by mesothelial cells, and is a tumor-associated antigen overexpressed on various malignant tumor cells, including malignant pleural mesothelioma (MPM)^41^. It is a serum marker used to monitor mesothelioma, and studies also show higher levels in PEs of MPM patients, but with many false positives from other malignant PEs^42,43^. An analysis of lung tumors showed that MSLN was also overexpressed in half of ADCs, in large cell carcinomas, and in SCC, but was absent in SCLC^43^. This was precisely observed in our study, based just on PE analysis, and is likely the reason why MSLN is one of the top markers for a general cancerous-PE classification.

ADA2, CXCL10, and WARS were the top proteins that distinguished the TB-PEs from all other PEs, and the only proteins that significantly discriminated the TB-PEs from the other-infectious-PEs. WARS is the interferon γ-inducible, cytoplasmic form of tryptophanyl-tRNA synthetase. WARS was increased in human macrophages infected with *Mycobacterium tuberculosis*, and in the serum of patients with active pulmonary TB, being one of the top three TB candidate biomarkers^44,45^. We show here, for the first time, that WARS is also higher specifically in TB-PEs, and, with an AUC of 0.832, it shows a high discriminating potential. CXCL10, also known as interferon γ-induced protein 10 kDa (IP-10), is a cytokine belonging to the CXC chemokine family^46^. It attracts monocytes and activated T lymphocytes to inflamed sites, and promotes selective enhancement of Th1 responses and increased IFN-γ production. CXCL10 has been reported elevated in TB effusions, and like ADA and IFN-γ, is indicative of acute disease and has been suggested as an additional marker for diagnosis^47–49^. Seven earlier studies in which CXCL10 concentration was measured by ELISA showed a mean sensitivity of 84%, and a specificity of 90% for discriminating TB-PEs^3^. Also at 90% specificity, our observed sensitivity is 71% for CXCL10 (AUC: 0.880), but classifier use raises these to 94% and 75%, respectively. ADA2 had the highest discriminating power (AUC: 0.881), and was the top scoring feature in the classifier. Adenosine deaminase (ADA), represented by two isoenzymes (ADA1 and ADA2), is involved in purine metabolism and catalyzes the deamination of adenosine to inosine, a signaling molecule that is central to lymphoid system development^50^. ADA1 constitutes the majority of intracellular ADA activity, while ADA2 is secreted specifically by the monocyte-macrophage cell system and T-lymphocytes, and is the predominant isoenzyme in TB effusions^50–52^. The utility of measuring total ADA activity for diagnosing pleural TB was first reported in 1978, and remains to this day the most qualified biomarker for TB-PEs^3^. Five meta-analyses show that pleural fluid ADA activity has a sensitivity of 92% and a specificity of 90%^3^. Measuring ADA2 activity has been shown to be superior to measuring total activity^51^, however, clinically used tests still measure total ADA activity. Our assay has the ability to easily distinguish the two isoenzymes, and we did not detect ADA1 in any of the PEs tested while targeting its three peptides. Although this could be an issue of the assay's limit of detection, it confirms that ADA2 is predominant in TB-PEs. ADA1 is also produced by neutrophils and occurs mostly in non-tuberculous PEs such as complicated parapneumonic effusions and empyemas, and is responsible for most false-positive cases in non-TB-PEs^3,51^. Although such PEs are usually easily distinguished clinically, and empyemas were thus excluded in our study, monitoring both isoenzymes could add diagnostic value.

Parapneumonic PEs are secondary to pneumonia or lung infection, and also require a rapid diagnosis as mortality is higher among patients with an associated PE^1^. The “gold standard” for diagnosing a pleural infection is the growth of an organism, however, 40% of aspirates are negative and this requires a significant amount of time, while results from quicker biochemical tests can be similar to those from malignant-PEs^2^. Seven infection/inflammation-related proteins in our assay, S100A9, VIM, LGALS1, APCS, MMP9, LDHA, and CRP, were elevated in TB-PEs and the other-infectious-PEs. The other-infectious-PEs did not have unique elevated markers, but S100A9, APCS, and CRP were the top significant proteins in contrast to what was observed for the TB-PEs. Additionally, low CXCL10, ADA2, and WARS, and higher HPX levels, effectively discriminate these from the TB-PEs. The classifier selected CRP, S100A9, and VIM, as the top features for discriminating the other-infectious-PEs, and could just as effectively discriminate this group (AUC: 0.863), as the TB-PEs (AUC: 0.859), or the all-infectious-PEs (AUC: 0.869). For comparison, predictive models based on clinical measurements of CRP, IL-6 and neutrophil/leukocyte cell counts diagnosed infectious PEs with AUCs of 0.898–0.909^53^.

S100A9 is a calcium binding protein that mainly exists as a S100A8/A9 heterodimer (calprotectin), which is secreted by neutrophils and monocytes, modulating inflammatory processes by stimulating leukocyte recruitment and inducing cytokine secretion, with potential as a diagnostic biomarker for inflammation associated diseases^54^. APCS (serum Amyloid P component, or SAP) is a plasma protein, which like CRP, belongs to the pentraxin family of calcium-dependent ligand binding proteins, and is associated with several pathogenic amyloid conditions^55^. It inhibits neutrophil movement into tissues, promotes the resolution of inflammation and fibrosis, promotes phagocytosis, and regulates macrophage differentiation^56^. Higher levels of S100A9 and APCS have been found in parapneumonic effusions (and also in TB-PEs for APCS) than in malignant (ADC) effusions^39^, and we have now confirmed this finding on a larger and more varied sample set. CRP is an acute-phase inflammatory protein, clinically utilized as a marker of infection and cardiovascular events^57^. It was elevated in infectious PEs, including tuberculous, parapneumonic and empyemas, when compared to malignant and transudative PEs^58–60^. A meta-analysis of 18 publications indicated a reasonable diagnostic ability of CRP levels for parapneumonic PEs (AUC: 0.88, 80% sensitivity, 82% specificity)^61^. VIM is a type III intermediate filament protein involved in maintaining cellular integrity, and other processes as cell attachment, migration, and signaling^62,63^. Classically, a marker of the epithelial-mesenchymal transition (EMT), it is associated with poor prognosis, and has been found overexpressed in various epithelial cancers, including lung cancer^62^. VIM was upregulated on the surface of human monocytes infected with *M. tuberculosis*, and as a ligand mediated their lysis by natural killer cells^44,64^. Although we observed VIM sporadically high in a number of cancerous-PEs, including for ADC, SCC, SCLC, mesothelioma and secondary cancers, the levels of VIM were more specifically elevated in the infectious-PEs, as we already previously observed^16^. After S100A9, the classifier picked VIM as the second top feature for the all-infectious-PE group. Rather than being a cancer marker, we suggest that VIM be classed as a marker of infection in PE, which is also in agreement with a study by Kanaji et al., where VIM did not discriminate malignant- from non-malignant-PEs^65^. LGALS1, MMP9, and LDHA were not as effective at discriminating the other-infectious-PEs from all PEs as the above markers, but they were still significantly elevated. LGALS1 (Galectin-1) is a lectin with anti-inflammatory functions^66,67^. Its overexpression has been reported in acute and chronic inflammation, but also in several tumor types including lung cancer, where it can influence tumor progression^67,68^. Mundt et al., showed overexpression of LGALS1 in ADC-related PEs when compared to mesothelioma-related PEs, while Javadi et al., found the opposite, with both claiming LGALS1 as a potential marker for mesothelioma^69,70^. We found higher expression of LGALS1 in three of the four mesothelioma PEs and in some cancer-PEs, but higher LGALS1 levels are more prominent in the infectious PEs. MMP9 (gelatinase B) stimulates or inhibits the process of degradation of the extracellular matrix (ECM), and its overexpression is associated with various cancers, as well as with acute and chronic inflammatory and infectious diseases^71^. MMP9 was increased in TB-PEs compared with transudates and malignant PEs, and was produced by epithelioid cells within granulomas^18,72,73^. In these earlier studies, no other infectious PEs were analyzed, while here we have shown that MMP9, in addition to TB-PEs, is also elevated in parapneumonic PEs. LDH is an essential cytosolic enzyme, reversibly converting pyruvate to lactate. It is a homo- or hetero-tetramer, forming five isoenzymes, that differ in their proportion of the LDHA and LDHB subunits, and tissue distribution^74^. LDH is released by damaged cells, and total serum LDH is a highly sensitive, but nonspecific marker of numerous clinical conditions^75^. LDH activity is used in the differentiation of transudate and exudate PEs, with exudates, especially complicated parapneumonic and TB-PEs, having higher LDH^1,76^. Our results confirmed this, and additionally revealed that the LDHA subunit predominates in the infectious-PEs, and is also higher in infectious-PEs than in cancerous-PEs, in contrast to LDHB.

Although often considered as ‘benign’ effusions, PEs related with heart, hepatic, and renal failure are associated with a 25–50% 1-year mortality rate^1^. The benign-PEs, although not possessing any unique elevated proteins and presenting the lowest discrimination potential, could still be effectively differentiated by lower levels especially of C9, LDHA, HPX, LDHB, and C3. This appears to be an effect of such PEs being mostly transudates with inherently lower plasma protein concentrations. Complement proteins C9 and C3 are clinically used as markers of inflammation, and were also reported as a potential serological marker of TB and as a marker of parapneumonic PEs, respectively^39,45^. We have shown they are better markers of a general exudate PE, and are lower in transudate PEs. SERPINA4, SERPINF1, and GSN were also suggested previously as potential serological and PE markers of TB or malignancy^39,45,77^, and despite detecting these proteins with strong signals using multiple peptides in all samples, we did not see significant indications for these as markers of any specific PE etiology.

In conclusion, we compared clinical and potential markers of PE etiology, side-by-side, using MS-based multiplexed targeted proteomics, on a PE set representative of a real clinical setting. We have determined the most promising proteins to be pursued in future prospective studies on clinical MS-based platforms. These include: CDH1, MUC1, THBS4, and MSLN for PEs related to cancer; ADA2, CXCL10, and WARS for TB-PEs; CRP, S100A9, and VIM for parapneumonic PEs; and C9, LDHA, HPX, LDHB, and C3 for benign-PEs. The assay and the developed classifier create a tool that is highly effective at confirming a diagnosis, especially for a cancerous- or TB-PE. Although, in general, the diagnostic performance of our biomarker panel did not outperform the currently used clinical tests, this type of multiplexed assay, measuring more than one-disease-marker in a single analysis, paves the way towards diagnosing multiple etiologies with a single laboratory test. We have revealed new information for many markers on their PE-related expression due to the greater variety of PEs studied, and we have shown that novel isoenzyme-specific information can be provided by MS. Such MS-based tests, based on minimally invasive and often medically required thoracentesis and PE sampling, performed early on, could quickly provide information-rich supportive guidance towards subsequent more- or less-invasive procedures required for a definite diagnosis and patient treatment.

## Supplementary Information

Supplementary Information.

Supplementary Table 3.

Supplementary Table 3.

## Data Availability

The MRM dataset generated and analyzed during the current study is available in the Panorama Public repository: https://panoramaweb.org/Robak-et-al-2021.url.
