# Supplementary material for: Diagnosing pleural effusions using mass spectrometry-based multiplexed targeted proteomics quantitating mid- to high-abundance markers of cancer, infection/inflammation and tuberculosis
Source: Sci Rep. 2022 Feb 23;12:3054. doi: 10.1038/s41598-022-06924-y (PMC8866415; doi:10.1038/s41598-022-06924-y)
Supplement: Supplementary file 1 — Supplementary Information. [file 41598_2022_6924_MOESM1_ESM.pdf]

Supplementary information

Diagnosing pleural effusions using mass spectrometry-based multiplexed targeted proteomics quantitating mid- to high-abundance markers of cancer, infection/inflammation and tuberculosis

Aleksandra Robak[1], Michał Kistowski[1], Grzegorz Wojtas[2], Anna Perzanowska[1], Tomasz Targowski[3], Agata Michalak[2], Grzegorz Krasowski[2], Michał Dadlez[1], *Dominik Domański[1]

[1]Mass Spectrometry Laboratory, Institute of Biochemistry and Biophysics - Polish Academy of Sciences, Warsaw, Poland.

[2]Mazovian Center of Pulmonary Disease and Tuberculosis Treatment, Otwock, Poland.

[3]Department of Geriatrics, National Institute of Geriatrics, Rheumatology and Rehabilitation, Warsaw, Poland.

*Corresponding author: dom.domanski@ibb.waw.pl

Table of contents

Supplementary Figure S1. 2

Supplementary Figure S2. 3

Supplementary Figure S3. 4

Supplementary Figure S4. 5

Supplementary Table S1. 6

Supplementary Table S2. 8

Supplementary Table S3. **Supplied as Excel file**

Supplementary Table S4. **Supplied as Excel file**

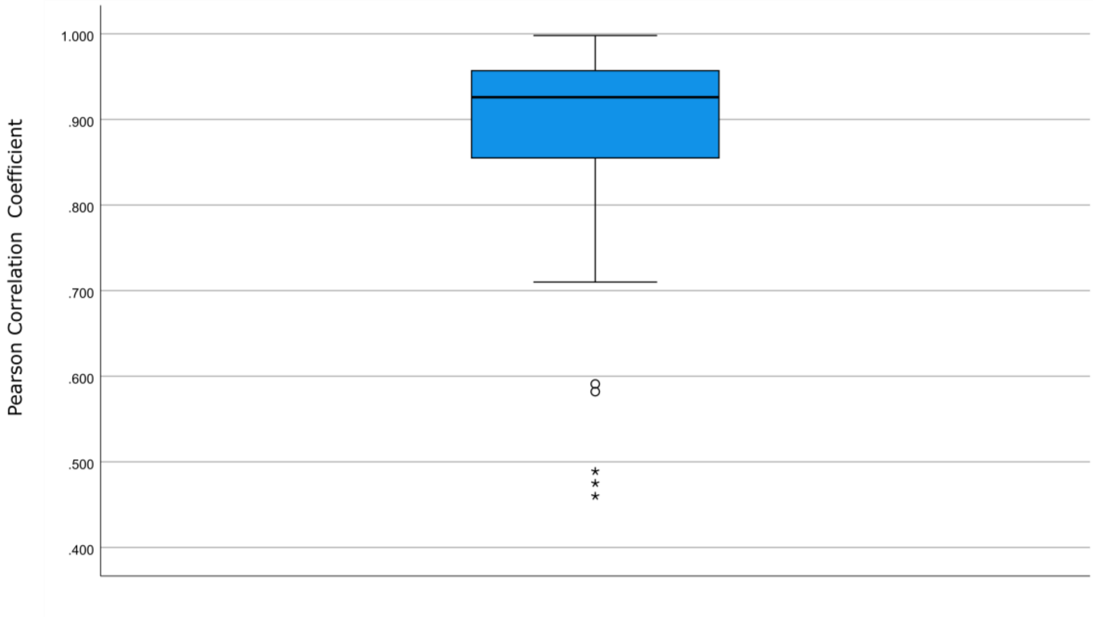

Supplementary Figure S1. Pearson correlation coefficients showing agreement of quantitative levels from multiple peptides used per protein in the 209 PE data set for proteins where multiple peptides were used (See Table 1). Box-plot indicates the median and quartiles, with whiskers indicating the 1.5 interquartile range. Outliers are identified by circles (‘out values’: between 1.5 IQR’s and 3 IQR’s) and stars (‘far out values’: >3 IQR’s). All values were used, showing a median Pearson correlation coefficient (R value) of 0.926 ± SD 0.124.

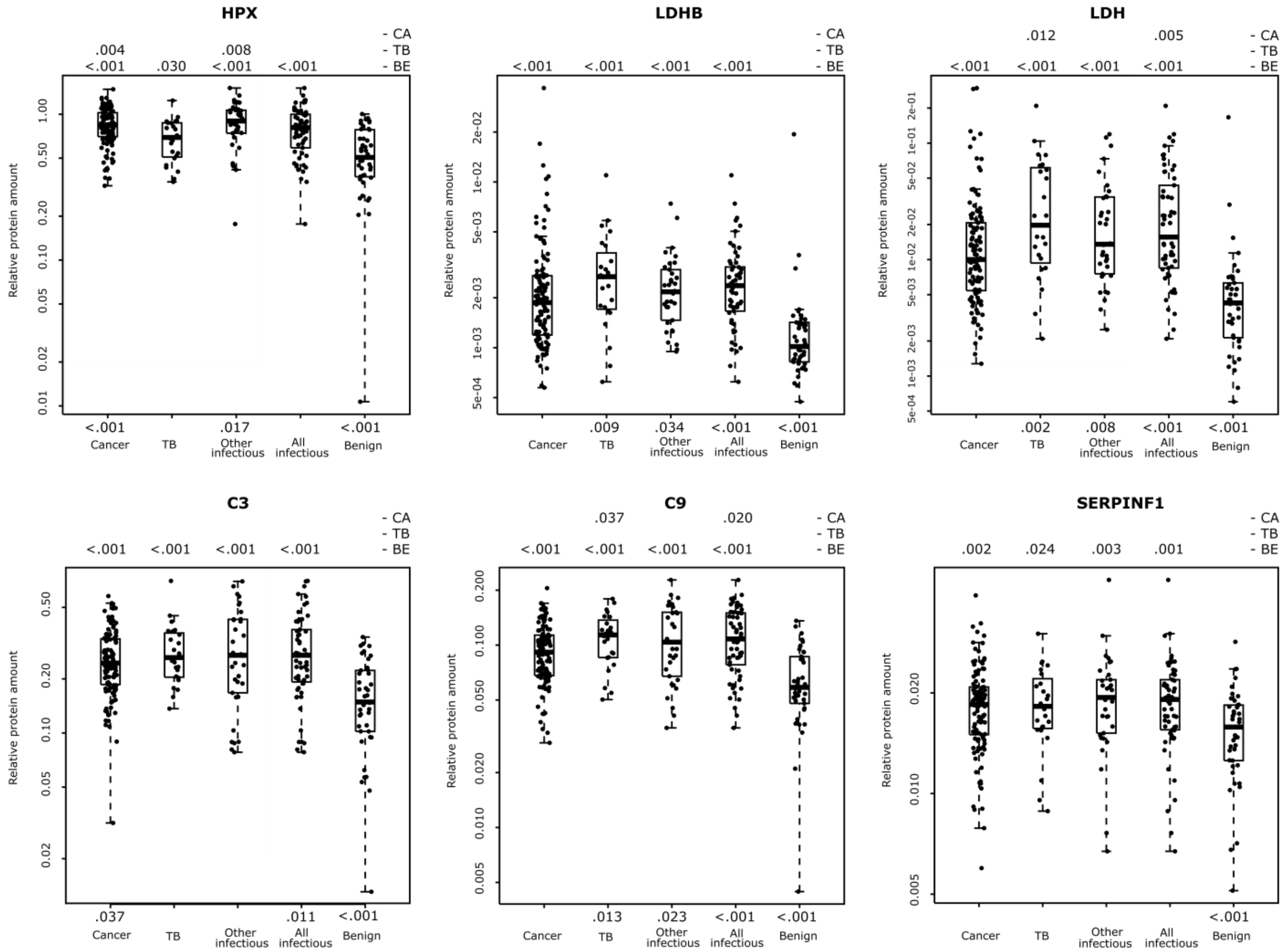

Supplementary Figure S2. Proteins significantly decreased in the benign-PEs. Relative protein amounts in 209 patient PEs. Any statistically significant comparisons (p-value ≤0.05, Mann-Whitney *U* test) are indicated above the graphs for the comparison of the cancerous-PEs (CA), TB-PEs (TB), or benign-PEs (BE) vs. cancerous-PEs, TB-PEs, other-infectious-PEs, or all-infectious-PEs (value above each group). Below the graphs are indicted any significant p-values for the comparison of all five PE groups independently vs. all other PEs combined. Box-plots indicate the median and quartiles, with whiskers indicating the 1.5 interquartile range.

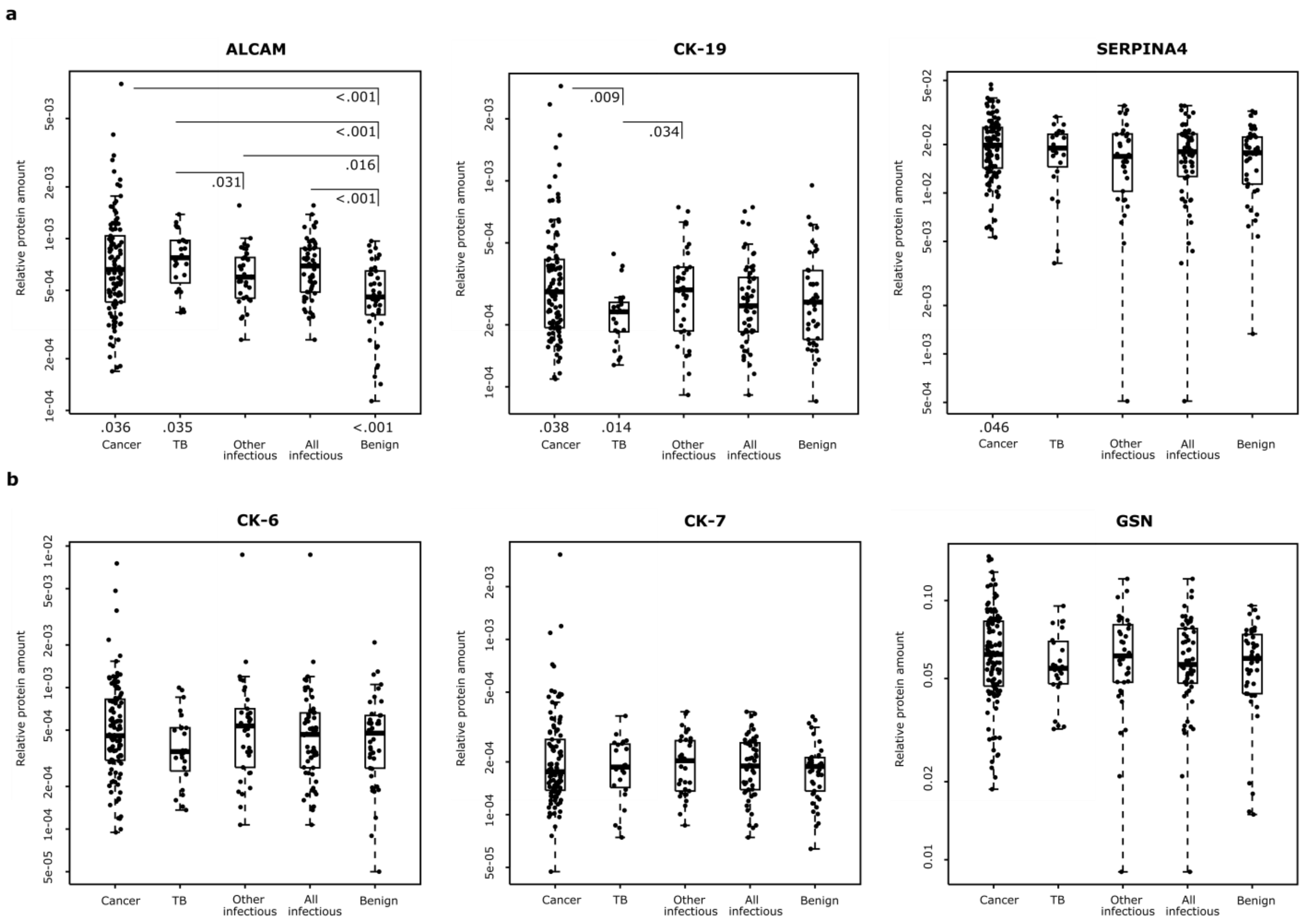

Supplementary Figure S3. Proteins significantly elevated in the cancerous-PEs with a lower discrimination ability (a), and non-significant proteins (b). Relative protein amounts in 209 patient PEs. Any statistically significant comparisons (p-value ≤0.05, Mann-Whitney *U* test) are indicated for individual PE groups by brackets, and below the graphs for any significant comparisons of all five PE groups independently vs. all other PEs combined. Box-plots indicate the median and quartiles, with whiskers indicating the 1.5 interquartile range.

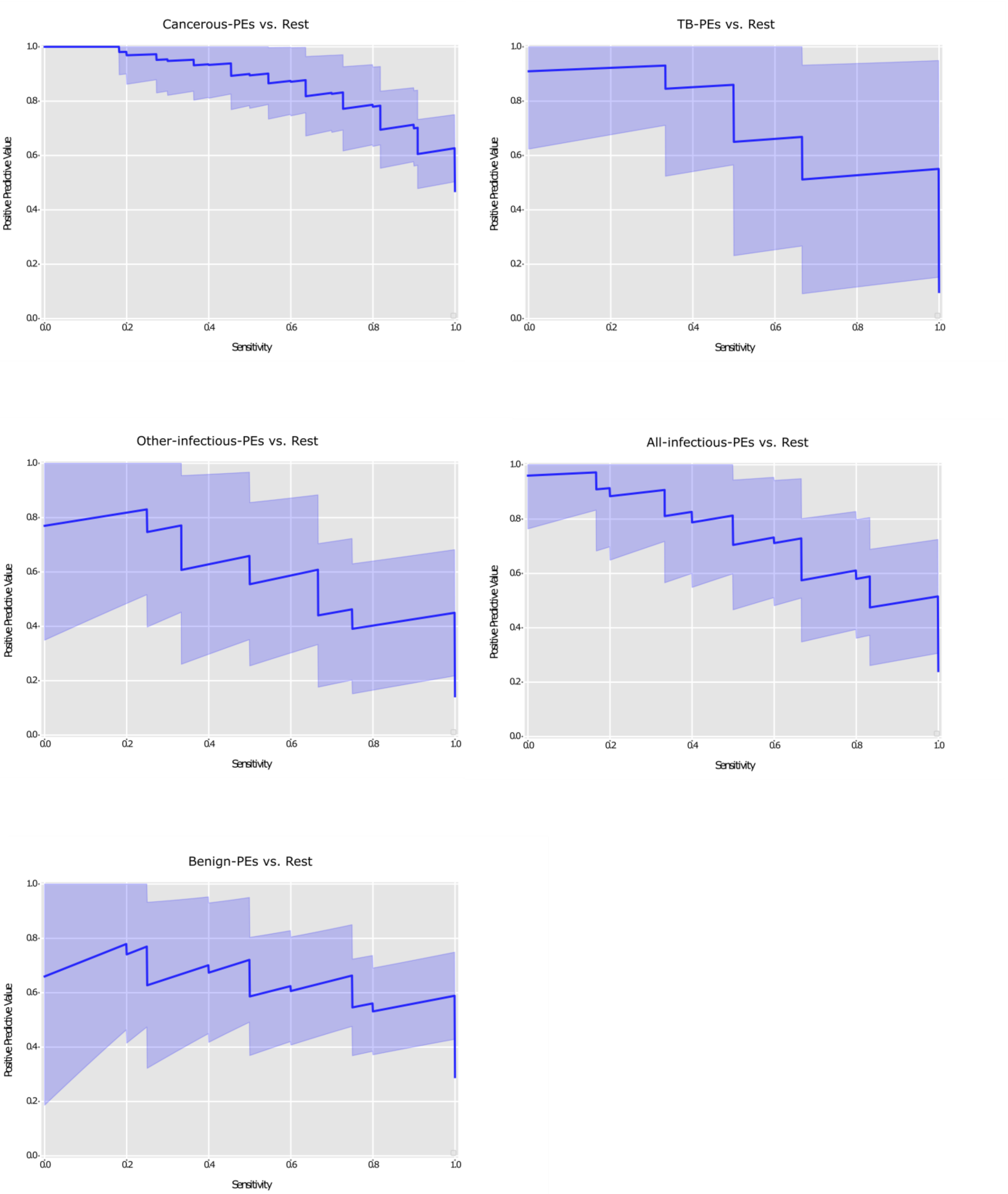

Supplementary Figure S4. The curves of Positive Predictive Value (PPV) vs. Sensitivity for each of the five classifications. Presented as mean PPV (blue line) ± standard deviation (violet).

Supplementary Table S1. Makeup of the PE sample set (a), demographic data of the study population (b) and flow-chart showing PEs included and excluded from the analysis (c). One hundred and fifty-eight PEs were excluded from the analysis: 65 purulent empyema PEs (frank pus, and therefore easily diagnosed), 55 PEs of unknown etiology, 8 PEs with two or more etiologies, and 30 benign-PEs (undefined transudate PEs, non-cancerous and non-infectious). Two hundred and nine PEs with a diagnosed etiology were taken for analysis. One hundred and nine PEs were from patients with a diagnosed form of cancer (cancerous-PEs), and included 82 lung cancers (65 non-small-cell lung carcinomas with 33 ADC and 22 SCC subtypes) and 27 secondary cancers. Fifty-eight PEs were from patients with infectious diseases (all-infectious PEs) and included 25 parapneumonic PEs, 24 tuberculous PEs (TB-PEs), 6 pleuritic PEs and 3 empyema PEs (non-purulent, i.e. no visual manifestation of pus). Forty-two PEs were from patients with non-cancerous and non-infectious ailments (benign-PEs), and included 27 heart failure-PEs, four transudates, three renal failure-PEs, two kidney failure-PEs, two arthritis-PEs, and one of each PE related with: pancreatitis, cirrhosis, pulmonary embolism, and post-trauma.

**a**		**b**		
**Cancer**	**109**		**Number of Patients**	**Median Age (yr)**
Lung cancer	82	**Cancer**		**67**
NSCLC	65	Female	42	
ADC	33	Male	67	
SCC	22	**Infectious**		**58**
SCLC	17	**Pneumonia**		**60**
Non-lung cancer	27	Female	5	
**Infectious**	**58**	Male	25	
Pneumonia	25	**Tuberculosis**		**55**
Tuberculosis	24	Female	1	
Pleurisy	6	Male	23	
Empyema	3	**Pleurisy**		**51**
**Benign**	**42**	Female	3	
Heart failure	27	Male	3	
Transudate	4	**Empyema**		**57**
Renal failure	3	Female	1	
Kidney failure	2	Male	2	
Arthritis	2	**Benign**		**70.5**
Pulmonary embolism	1	Female	10	
Pancreatitis	1	Male	32	
Cirrhosis	1			
Trauma	1			

c

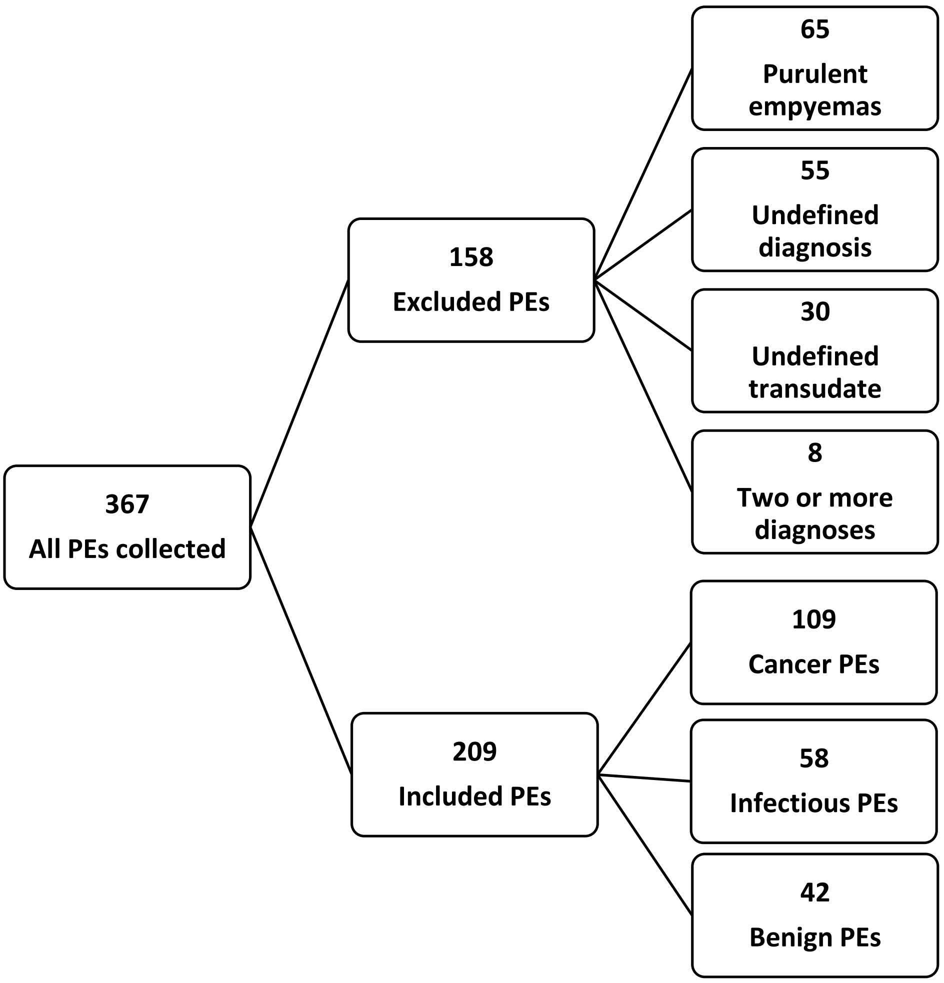

367
All PEs collected
158
Excluded PEs
209
Included PEs
65
Purulent empyemas
55
Undefined diagnosis
30
Undefined transudate
8
Two or more diagnoses
109
Cancer PEs
58
Infectious PEs
42
Benign PEs

Supplementary Table S2. Tested protein markers but not detected in PEs out of fifty-three proteins assessed in this study. The fifty-three proteins consist of markers currently used in the clinic, and putative markers proposed in the research literature, which have shown potential in the diagnosis or classification of PEs, of cancer samples, or disease from plasma/serum testing, using clinically established biochemical or antibody-based assays, or using research techniques such as global and targeted MS or antibody-based single- and multi-plexed methods. Twenty-nine proteins (ADA1 and ADA2 (representing ADA), C3, C9, CEA/CEACAM5, CK-6, CK-7, CK-8, CK-14, CK-17, CK-18, CK-19, CRP, GRP, IFNG, LDHA, LDHB, and LDHC (representing LDH), MSLN, MUC-1/CA 15-3, MUC-16/CA-125, NSE, CDH1, MMP9, EPCAM, VIM, WFDC2/HE4, SPB3/SCCA-1 and SPB4/SCCA-2 (representing SCCA)) represent clinical laboratory tests. Thirty-two, thirteen, and eight proteins are verified or putative cancer, TB, or infection/inflammation markers, respectively.

**Protein name (abbreviation used in text)**<br>(other names)<br>[gene name, UniProt identifier]	**Peptide measured by MRM**<br>(Peptide position in protein)	**Reported concentration range in PEs**	**Description**
**Carcinoembryonic antigen-related cell adhesion molecule 5 (CEA/CEACAM5)**<br>(Carcinoembryonic antigen, CEA)<br>[CEACAM5, P06731]<br>Length: 702 aa, Mass (Da): 76,795	QIIGYVIGTQQATPGPAYSGR (78-98)<br>SDLVNEEATGQFR (127-139)<br>NDTASYK (208-214)<br>LQLSNDNR (369-376)<br>INGIPQQHTQVLFIAK (629-644)	2 - 660<br>ng/ml	Potential marker of malignant PE[1,2]. Clinical serum marker for monitoring tumors of digestive organs[1].
**Dipeptidyl peptidase 4 (DPP4)**<br>[DPP4, P27487]<br>Length: 766 aa, Mass (Da): 88,279	FRPSEPHFTLDGNSFYK (357-373)<br>VLEDNSALDK (493-502)<br>LGTFEVEDQIEAAR (598-611)	154 - 264<br>ng/ml	Potential marker of malignant PE[3].
**DNA replication complex GINS protein PSF3 (GINS3)**<br>[GINS3, Q9BRX5]<br>Length: 216 aa, Mass (Da): 24,535	LGAFFLER (43-50)<br>SAGAETDNAVPQGSK (51-65)<br>LELPLWLAK (66-74)<br>TVFSADPNVVDLHK (98-111)<br>IMDSSQNAYNEDTSALVAR (149-167)<br>GQASQITASNLVQNYK (192-207)	NA	Potential prognostic immunohistochemical marker for Lung ADC[4].
**Epithelial cell adhesion molecule (EPCAM)**<br>[EPCAM, P16422]<br>Length: 314 aa, Mass (Da): 34,932	LAVNCFVNNNR (34-44)<br>QCQCTSVGAQNTVICSK (45-61)<br>EKPYDSK (154-160)<br>TQNDVDIADVAYYFEK (203-218)<br>APEFSMQGLK (256-265)	NA	Potential marker of malignant PE[5,6]. Tumor marker used in pathology for identifying Lung ADC[5].
**Gamma enolase (NSE)**<br>(neuron specific enolase, NSE)<br>[ENO2, P09104]<br>Length: 434 aa, Mass (Da): 47,269	GNPTVEVDLYTAK (16-28)<br>AVDHINSTIAPALISSGLSVVEQEK (65-89)<br>YITGDQLGALYQDFVR (270-285)<br>IEEELGDEAR (413-422)	9 - 91<br>ng/ml	Potential marker of malignant PE[2]. Clinical serum SCLC diagnostic marker[2].
**Gastrin-releasing peptide (GRP)**<br>(GRP)<br>[GRP, P07492]<br>Length: 148 aa, Mass (Da): 16,213	AVPLPAGGGTVLTK (23-36)<br>VPLPAGGGTVLTK (24-36)<br>STGESSSVSER (54-64)<br>NLLGLIEAK (83-91)<br>NHQPPQPK (95-102)<br>ALGNQQPSWDSEDSSNFK (103-120)<br>LSAPGSQR (131-138)	NA	Potential marker of malignant PE[7]. Clinical serum SCLC diagnostic marker[8,9].
**Hepatocyte growth factor receptor (MET)**<br>[MET, P08581]<br>Length: 1,390 aa, Mass (Da): 155,541	EVFNILQAAYVSKPGAQLAR (312-331)<br>GDLTIANLGTSEGR (448-461)<br>LNSELNIEWK (903-912)	133 - 528<br>ng/ml	Potential marker of malignant PE[3].
**Kallikrein-12 (KLK12)**<br>[KLK12, Q9UKR0]<br>Length: 248 aa, Mass (Da): 26,734	NSQPWQVGLFEGTSLR (31-46)<br>LGEHSLSQLDWTEQIR (72-87)	NA	Potential marker of malignant pleural mesothelioma PE[10].
**Keratin, type I cytoskeletal 14 (CK-14)**<br>**[KRT14,** P02533]<br>Length: 472 aa, Mass (Da): 51,561	APSTYGGGLSVSSSR (42-56)<br>DAEEWFFTK (301-309)	0 – 4<br>ng/mg protein	Tumor marker used in pathology for differentiation of lung cancer[11].
**Keratin, type I cytoskeletal 16 (CK-16)**<br>[KRT16, P08779]<br>Length: 473 aa, Mass (Da): 51,268	APSTYGGGLSVSSR (42-55)<br>DAETWFLSK (303-311)	0 – 9<br>ng/mg protein	Potential immunohistochemical marker for SCC[12]
**Keratin, type I cytoskeletal 17 (CK-17)**<br>[KRT17, Q04695]<br>Length: 432 aa, Mass (Da): 48,106	ALEEANTELEVK (104-115)<br>AAPGVDLSR (243-251)<br>ASLEGNLAETENR (322-334)<br>TIVEEVQDGK (410-419)	0 – 5<br>ng/mg protein	Tumor marker used in pathology for differentiation of lung cancer[11]. Potential marker of malignant PE[13].
**Mucin 16 (MUC-16/CA-125)**<br>(MUC-16, Ovarian cancer-related tumor marker CA-125)<br>[MUC16, Q8WXI7]<br>Length: 14,507 aa, Mass (Da): 1,519,175	DSLFINGYAPQNLSIR (14184-14199)<br>ALFSSNLDPSLVEQVFLDK (14266-14284)<br>VAIYEEFLR (14405-14413)	7 - 309<br>U/ml	Potential marker of malignant PE[1]. Clinical serum tumor marker for monitoring ovarian cancer[1].
**Receptor-type tyrosine-protein phosphatase F (PTPRF)**<br>[PTPRF, P10586]<br>Length: 1,907 aa, Mass (Da): 212,879	YSIGGLSPFSEYAFR (374-388)<br>VLAFTAVGDGPPSPTIQVK (488-506)<br>TGEGFIDFIGQVHK (1811-1824)	77 - 166<br>ng/ml	Potential marker of malignant PE[3].
**Serpin B3 (SPB3/SCCA-1)**<br>(Squamous cell carcinoma antigen 1, SCCA-1)<br>[SPB3, P29508]<br>Length: 390 aa, Mass (Da): 44,565	VLHFDQVTENTTGK (56-69)<br>FYQTSVESVDFANAPEESR (126-144)<br>QYTSFHFASLEDVQAK (215-230)<br>GLVLSGVLHK (322-331)	NA	Clinical serum tumor marker for monitoring SCC[8,14].

**Serpin B4 (SPB4/SCCA-2)**<br>(Squamous cell carcinoma antigen 2, SCCA-2)<br>[SPB4, P48594]<br>Length: 390 aa, Mass (Da): 44,854	VLHFDQVTENTTEK (56-69)<br>FYQTSVESTDFANAPEESR (126-144)<br>QYNSFNFALLEDVQAK (215-230)<br>ETCVDLHLPR (277-286)<br><br>Peptides in common to SPB3, SPB4:<br>FMFDLFQQFR SPB3, SPB4 (11-20)<br>STDAYELK SPB3, SPB4 (95-102)<br>INSWVESQTNEK SPB3, SPB4 (147-158)<br>TNSILFYGR SPB3, SPB4 (378-386)	NA	Clinical serum tumor marker for monitoring SCC[8,14].
**Trefoil factor 3 (TFF3)**<br>[TFF3, Q07654]<br>Length: 94 aa, Mass (Da): 10,181	VDCGYPHVTPK (54-64)<br>GCCFDSR (70-76)	NA	Potential immunohistochemical marker for differentiation of ADC and SCC[15].
**WAP four-disulfide core domain protein 2 (WFDC2/HE4)**<br>(Epididymal secretory protein E4, HE4)<br>[WFDC2, Q14508]<br>Length: 124 aa, Mass (Da): 12,993	CCSAGCATFCSLPNDK (61-76)<br>EGSCPQVNINFPQLGLCR (77-94)<br>DQCQVDSQCPGQMK (95-108)	NA	Potential serological marker of lung cancer[8].<br>Clinical serum marker for monitoring ovarian cancer[8].
**Adenosine deaminase (ADA1)**<br>[ADA, P00813]<br>Length: 363 aa, Mass (Da): 40,764	VELHVHLDGSIKPETILYYGR (12-32)<br>YSPHLLANSK (102-111)<br>LGHGYHTLEDQALYNR (236-251)	NA	Clinically measured total ADA activity in PE is a marker of TB pleuritis[16]
**Cathepsin B (CTSB)**<br>[CTSB, P07858]<br>Length: 339 aa, Mass (Da): 37,822	NGPVEGAFSVYSDFLLYK (246-263)	NA	Potential marker of TB-PE[10].
**Interferon gamma (IFNG)**<br>(IFN-gamma)<br>[IFNG, P01579]<br>Length: 166 aa, Mass (Da): 19,348	YFNAGHSDVADNGTLFLGILK (37-57)<br>IMQSQIVSFYFK (67-78)<br>LTNYSVTDLNVQR (118-130)<br>AIHELIQVMAELSPAAK (132-148)	4 - 142 pg/ml	Potential marker of TB-PE[17–19].<br>Clinical serum and PE marker for latent TB[19].
**Interleukin-33 (IL33)**<br>[IL33, O95760]<br>Length: 270 aa, Mass (Da): 30,759	VLLSYYESQHPSNESGDGVDGK (159-180)<br>DFWLHANNK (191-199)<br>EHSVELHK (200-207)<br>TDPGVFIGVK (234-243)<br>DNHLALIK (244-251)<br>VDSSENLCTENILFK (252-266)	13 - 23 ng/l	Potential marker of TB-PE[20].
**Perforin-1 (PRF1)**<br>[PRF1, P14222]<br>Length: 555 aa, Mass (Da): 61,377	FVPGAWLAGEGVDVTSLR (37-54)<br>LISNYGTHFIR (215-225)<br>ALSQYLTDR (363-371)<br>LFFGGQELR (441-449)<br>TSTVWDNNNPIWSVR (450-464)<br>LDFGDVLLATGGPLR (465-479)	3 - 8 ng/ml	Potential marker of TB-PE[21].
**Tumor necrosis factor receptor superfamily member 6B (TNFRSF6B)**<br>[TNFRSF6B, O95407]<br>Length: 300 aa, Mass (Da): 32,680	VAETPTYPWR (30-39)<br>LVCAQCPPGTFVQRPCR (47-63)<br>DSPTTCGPCPPR (65-76)<br>VPGAEECER (205-213)<br>LLQALEAPEGWGPTPR (232-247)<br>LTELLGAQDGALLVR (261-275)	8 - 17 ng/ml	Potential marker of TB-PE[21].
**L-lactate dehydrogenase C chain (LDHC)**<br>[LDHC, P07864]<br>Length: 332 aa, Mass (Da): 36,311	LIEDDENSQCK (12-22)<br>DLADELALVDVALDK (43-57)<br>IVIVTAGAR (91-99)<br>SIIPAIVHYSPDCK (119-132)<br>SAETLWNIQK (319-328)	NA	Clinically used for exudative and transudative PE classification based on Light's Criteria[22]. Clinical serum marker of inflammation[23].

References

1. Porcel, J. M. *et al.* Use of a panel of tumor markers (carcinoembryonic antigen, cancer antigen 125, carbohydrate antigen 15-3, and cytokeratin 19 fragments) in pleural fluid for the differential diagnosis of benign and malignant effusions. *Chest* **126**, 1757–1763 (2004).

2. Lee, J. H. & Chang, J. H. Diagnostic utility of serum and pleural fluid carcinoembryonic antigen, neuron-specific enolase, and cytokeratin 19 fragments in patients with effusions from primary lung cancer. *Chest* **128**, 2298–2303 (2005).

3. Liu, P. J. *et al.* In-depth proteomic analysis of six types of exudative pleural effusions for nonsmall cell lung cancer biomarker discovery. *Mol. Cell. Proteomics* **14**, 917–932 (2015).

4. Tauchi, S. *et al.* Psf3 is a prognostic biomarker in lung adenocarcinoma: A larger trial using tissue microarrays of 864 consecutive resections. *Eur. J. Cardio-thoracic Surg.* **50**, 758–764 (2016).

5. Porcel, J. M. *et al.* Epithelial cell adhesion molecule (EpCAM) from pleural fluid cell lysates is a highly accurate diagnostic biomarker of adenocarcinomatous effusions. *Respirology* **24**, 799–804 (2019).

6. Roca, E. *et al.* Detection of EpCAM-positive microparticles in pleural fluid: A new approach to mini-invasively identify patients with malignant pleural effusions. *Oncotarget* **7**, 3357–3366 (2016).

7. Shijubo, N. *et al.* Elevated Progastrin-Releasing Peptide(31-98) Concentrations in Pleural Effusions due to Small-Cell Lung Carcinoma. *Respiration* **63**, 106–110 (1996).

8. Korkmaz, E. T. *et al.* Triple test with tumor markers CYFRA 21.1, HE4, and ProGRP might contribute to diagnosis and subtyping of lung cancer. *Clin. Biochem.* **58**, 15–19 (2018).

9. Cavalieri, S. *et al.* Clinical implications for pro-grp in small cell lung cancer. A single center experience. *Int. J. Biol. Markers* **33**, 55–61 (2018).

10. Porcel, J. M., Esquerda, A., Martínez-Alonso, M., Bielsa, S. & Salud, A. Identifying thoracic malignancies through pleural fluid biomarkers: A predictive multivariate model. *Med. (United States)* **95**, 1–8 (2016).

11. Chu, P. G. & Weiss, L. M. Keratin expression in human tissues and neoplasms. *Histopathology* **40**, 403–439 (2002).

12. Moll, R., Divo, M. & Langbein, L. The human keratins: Biology and pathology. *Histochem. Cell Biol.* **129**, 705–733 (2008).

13. Perzanowska, A. *et al.* An MRM-Based Cytokeratin Marker Assay as a Tool for Cancer Studies: Application to Lung Cancer Pleural Effusions. *Proteomics - Clin. Appl.* **12**, 1–13 (2018).

14. Uemura, Y. *et al.* Circulating serpin tumor markers SCCA1 and SCCA2 are not actively secreted but reside in the cytosol of squamous carcinoma cells. *Int. J. Cancer* **89**, 368–377 (2000).

15. Wang, X. N. *et al.* Trefoil Factor 3 as a Novel Biomarker to Distinguish between Adenocarcinoma and Squamous Cell Carcinoma. *Med. (United States)* **94**, 1–10 (2015).

16. Porcel, J. M., Esquerda, A. & Bielsa, S. Diagnostic performance of adenosine deaminase activity in pleural fluid: A single-center experience with over 2100 consecutive patients. *Eur. J. Intern. Med.* **21**, 419–423 (2010).

17. Keng, L. T. *et al.* Evaluating pleural ADA, ADA2, IFN-γ and IGRA for diagnosing tuberculous pleurisy. *J. Infect.* **67**, 294–302 (2013).

18. Chen, K. Y. *et al.* Novel biomarker analysis of pleural effusion enhances differentiation of tuberculous from malignant pleural effusion. *Int. J. Gen. Med.* **9**, 183–189 (2016).

19. Porcel, J. M. Tuberculous pleural effusion. *Lung* **187**, 263–270 (2009).

20. Xuan, W. X., Zhang, J. C., Zhou, Q., Yang, W. B. & Ma, L. J. IL-33 levels differentiate tuberculous pleurisy from malignant pleural effusions. *Oncol. Lett.* **8**, 449–453 (2014).

21. Shu, C. C. *et al.* Diagnostic role of inflammatory and anti-inflammatory cytokines and effector molecules of cytotoxic T lymphocytes in tuberculous pleural effusion. *Respirology* **20**, 147–154 (2015).

22. Porcel, J. M. & Light, R. W. Diagnostic approach to pleural effusion in adults. *Am. Fam. Physician* **73**, 1211–1220 (2006).

23. Erez, A. *et al.* Diagnostic and prognostic value of very high serum lactate dehydrogenase in admitted medical patients. *Isr. Med. Assoc. J.* **16**, 439–443 (2014).
